# Mapping chromatin remodelling in glioblastoma identifies epigenetic regulation of key molecular pathways and novel druggable targets

**DOI:** 10.1186/s12915-025-02127-9

**Published:** 2025-02-07

**Authors:** Claire Vinel, James Boot, Weiwei Jin, Nicola Pomella, Alexandra Hadaway, Charles Mein, Nicolae Radu Zabet, Silvia Marino

**Affiliations:** 1 Brain Tumour Research Centre, Faculty of Medicine and Dentistry, Blizard Institute, Queen Mary University London, London, UK; 2 Genome Centre, Faculty of Medicine and Dentistry, Blizard Institute, Queen Mary University London, London, UK; 3 Faculty of Medicine and Dentistry, Blizard Institute, Queen Mary University London, London, UK; 4 Barts Brain Tumour Centre, Faculty of Medicine and Dentistry, Blizard Institute, Queen Mary University London, London, UK

**Keywords:** Glioblastoma, Cell of origin, Epigenetics, Chromatin remodelling, Druggable targets

## Abstract

**Background:**

Glioblastoma is the most common and aggressive malignant brain tumour in the adult population and its prognosis is dismal. The heterogeneous nature of the tumour, to which epigenetic dysregulation significantly contributes, is among the main therapeutic challenges of the disease.

**Results:**

We have leveraged SYNGN, an experimental pipeline enabling the syngeneic comparison of glioblastoma stem cells and expanded potential stem cell (EPSC)-derived neural stem cells to identify regulatory features driven by chromatin remodelling specifically in glioblastoma stem cells.

**Conclusions:**

We show epigenetic regulation of the expression of genes and related signalling pathways contributing to glioblastoma development. We also identify novel epigenetically regulated druggable target genes on a patient-specific level, including SMOX and GABBR2.

**Supplementary Information:**

The online version contains supplementary material available at 10.1186/s12915-025-02127-9.

## Background

Glioblastoma is the most common and aggressive intrinsic malignant brain tumour in the adult population. It is incurable, and patients’ survival after diagnosis rarely exceeds 15 months with relapses occurring in all patients. Despite significant research efforts, specifically in characterising the genomic, epigenomic and molecular factors driving its development and recurrence, standard therapy including maximal safe surgical resection, radiotherapy and chemotherapy has not changed in almost two decades.

The heterogeneous nature of the tumour, both intra-tumoural and inter-patient, is among the main therapeutic challenges of the disease. At an intra-tumoural level, a subpopulation of cells called glioblastoma stem/initiating cells (GIC) has been identified from their ability to self-renew and give rise to a fully differentiated tumour upon xenotransplantation [1]. GIC significantly contribute to resistance to radiotherapy and chemotherapy [2], hence playing a crucial role in recurrence [3]. Compelling evidence support an origin of GIC from neural stem cells (NSC), self-renewing and multipotent cells driving brain development and homeostasis [1], albeit also displaying distinct cellular and molecular features that contribute to tumour growth. At the inter-patient level, distinct genetic changes characterise the tumours occurring in different patients and various subclassification of glioblastoma have been described [4], which however have had only a minor impact on identifying new therapies and related biomarkers so far. The contribution of epigenetic deregulation to inter-patient heterogeneity has been demonstrated, including differential methylation at the promoter of the MGMT gene which is to date the only biomarker predicting drug response in glioblastoma patients [5]. However, despite significant efforts in characterising DNA methylation [6–8] and microRNA [9] profiles in glioblastoma, chromatin remodelling and histone modifications remain less explored; hence, we still lack a global understanding of the regulatory mechanisms controlling gene expression programmes in this tumour.

Modifications of histones, particularly the N-terminal tails of histone 3 (H3) at lysin residues, influence chromatin accessibility and gene expression [10]. Acetylation of lysine 27 (H3K27ac), tri and monomethylation of lysine 4 (H3K4me3 and H3K4me1) and lysine 79 (H3K79me3), as well as trimethylation of lysine 36 (H3K36me3) are hallmarks of active chromatin, whilst methylation of lysine 9 (H3K9me3) and 27 (H3K27me3) are found at condensed/silent chromatin regions [11]. H3K27ac and H3K4me1 marks have been linked to functional enhancers in different cell types [12, 13], whilst regions with co-localisation of H3K4me3 and H3K27me3, known as “bivalent domains”, are found mostly in embryonic stem cells [14]. Mapping H3K27ac deposition in glioblastoma cell lines and tissue biopsies as well as in normal brain tissue revealed transcriptionally active chromatin with implications for core oncogenic dependency on super-enhancer-driven transcription factors and long noncoding RNAs [15]. Upregulation of the histone demethylase KDM6 in GIC led to redistribution of H3K27me3 and induction of treatment resistance through acquisition of a slow cycling state [16]. Maintenance of an undifferentiated state was shown to be achieved via bivalent modulation at lineage-specific genes [17, 18] in a highly interconnected network regulated by WNT, SHH and HOX developmental pathways [17]. Analysis of chromatin accessibility, histone modifications (H3K4me3, H3K27ac and H3K27me3), DNA methylation and gene expression revealed a regulatory connection between FOXM1 and ANXA2R in gliomagenesis [19]. The analysis of the distribution of H3K9ac and H3K9me3 in GIC as compared to differentiated tumour cells showed that GIC displayed an open and highly dynamic chromatin structure with loss of clustered H3K9me3 and concomitant aberrant H3K9 hyperacetylation at promoters linked to DNA damage response (DDR), thus demonstrating that the H3K9me3–H3K9ac equilibrium is crucial for GIC viability [20]. Integration of active chromatin landscapes with gene expression revealed novel transcriptional regulatory circuits, super-enhancers and transcription factors that regulate GSC identity and intertumoral diversity [21]. Furthermore, characterisation of the promoter-enhancer interactome and regulatory landscape of glioblastoma revealed profound rewiring of promoter-enhancer interactions, chromatin accessibility and redistribution of histone marks in this tumour [22].

Here, we have leveraged SYNGN, an experimental pipeline enabling the syngeneic comparison of GIC and expanded potential stem cell (EPSC)-derived NSC (iNSC) [23] to identify regulatory features driven by chromatin remodelling specifically in glioblastoma stem cells. We show multifactorial epigenetic regulation of the expression of genes and related signalling pathways contributing to glioblastoma development. We also identify novel epigenetically regulated druggable target genes on a patient-specific level, which could be further developed for future translational approaches to tackle this neoplasm.

## Results

### Differential analysis of histone modifications in neoplastic and normal stem cells reveals epigenetic regulation of genes and molecular pathways involved in glioblastoma pathogenesis

To interrogate the global epigenetic landscape across the SYNGN cohort of 10 paired GIC/iNSC lines [23], we performed genome-wide chromatin immunoprecipitation sequencing (ChIP Seq) for activating and repressing histone modifications (HM), including H3K4me3, H3K27ac, H3K36me3 and H3K27me3 (Additional File 1: Fig.S1a) [24]. H3K4me3 is predominantly enriched at promoters and transcriptional start sites (TSS) of expressed genes, H3K27ac is enriched at typical enhancer and super-enhancer regions, H3K36me3 is an elongation marker enriched in gene bodies and the PcG-catalysed H3K27me3 is involved in silencing gene expression [25]. GIC datasets showing low number of peaks (< 5000) (2 tracks: GIC54 H3K36me3 and GIC61 H3K27me3, Additional File 1: Fig.S1b) or displaying a profile clustering apart from other similar HM tracks (1 track: GIC19 H3K27ac Additional File 1: Fig.S1c) were considered technical failures and excluded from further analysis. Hierarchical clustering of all retained tracks shows distinct clusters in both iNSC (Additional File 1: Fig.S2a) and GIC (Additional File 1: Fig.S2b) for H3K27me3 and H3K36me3, but not for H3K4me3 and H3K27ac tracks in keeping with the expected overlapping distribution of these HM across the genome [26]. Importantly, principal component analysis (PCA) of ChIP Seq data of GIC and iNSC showed a clear separation between GIC and iNSC for each HM (Additional File 1: Fig.S3a) and correlation heatmaps of differentially bound sites highlighted differences between GIC and iNSC (Additional File 1: Fig.S3b).

**Fig. 1:**
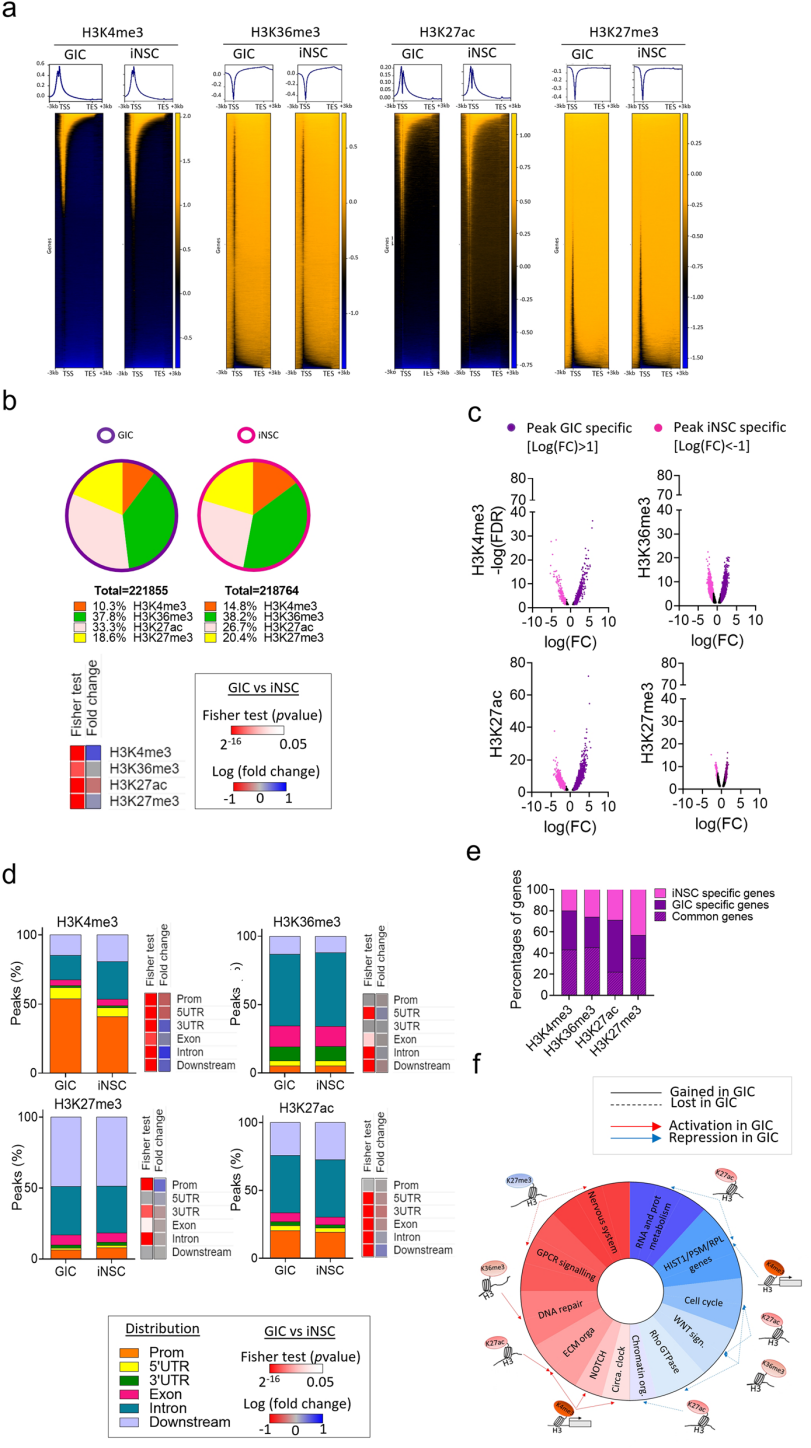
Mapping activating and repressing histone modifications in human GIC as compared to syngeneic iNSC. **a** Average heat map of ChIP Seq dataset around transcription start sites (TSS) and annotated genes for the 10 patients. Colours show read density. **b** Pie charts show proportion of peaks linked to each histone modification (HM) in GIC (left) and iNSC (right) in all patients. Heatmaps represent Fisher’s exact test statistical analysis and fold change of GIC ChIP-peaks upon iNSC ChIP-peaks for each HM. **c** Volcano plots represent the comparative analysis of differentially bound sites between GIC and iNSC for the four HM in all patients. Only significantly differentially bound sites are shown (FDR < 0.05). Results are represented as differential log fold change of GIC upon iNSC [Log2(GIC)-Log2(iNSC). Bound sites only found in GIC are shown as purple dots (log(FC) > 1) and bound sites found only in iNSC are shown as pink dots (log(FC)M < − 1). Bound sites with a − 1 < log(FC) < 1 are shown as black dots. **d** Distribution of ChIP Seq peaks’ genomic annotations for each HM GIC and iNSC. Heatmap represent Fisher’s exact test statistical analysis and fold change of ChIP Seq peaks between GIC and iNSC. **e** Percentages of genes common, only found in GIC (GIC specific) and only found in iNSC (iNSC specific) in each HM. **f** Schematic representation of HM redistribution in GIC as compared to iNSC and their targeted pathways. Red and blue shades in the donut represent activated and repressed signalling pathways, respectively

To ensure we focus on fundamental differences between the normal and neoplastic chromatin context in glioblastoma, we identified common epigenetic features shared between all 10 patients (Fig. 1a). Analysis of the number of peaks of each single HM (SHM analysis) showed quantitative differences with a higher proportion of total H3K27ac peaks (33.3% vs 26.7%) and a smaller proportion of H3K4me3 peaks (10.3% vs 14.8%) in GIC and iNSC (Fig. 1b) as well as high overlapping of peaks between GIC and iNSC (Additional File 1: Fig.S3c). Analysis of sites differentially enriched in each HM indicated differences between GIC and iNSC for all HM (Fig. 1c). Interestingly, genomic region annotation of the ChIP Seq peaks for each HM revealed also qualitative differences between GIC and iNSC most strikingly in H3K4me3 enrichment at promoter and 5′ UTR regions and in H3K27me3 depletion at promoter regions in GIC as compared to iNSC (Fig. 1d). We then focused on the genomic regions with an expected functional impact for each HM: promoters for H3K4me3; intron and exon for H3K36me3; promoters and intron/exon for H3K27me3 and H3K27ac. Comparative analysis of the genes identified in these genomic regions in GIC and iNSC revealed that only 22 and 35% were common for H3K27ac and H3K27me3 respectively, indicating an important chromatin remodelling in GIC, which was more pronounced for these HM given that significantly more genes identified in regions with H3K4me3 and H3K36me3 peaks (43 and 45%) were common between iNSC and GIC (Fig. 1e).

Gene set enrichment analysis (GSEA) identified significantly enriched pathways (FDR < 0.05) activated or repressed in GIC (Fig. 1f) which are known to be deregulated in glioblastoma, including enrichment for neuronal system, GPCR signalling, DNA repair, extra-cellular matrix organisation, NOTCH signalling and circadian clock for genes with an activating HM in GIC (Fig. 1f and Additional File 1: Fig.S4a). Conversely, genes with a repressing HM in GIC were mostly involved in metabolism of RNA and protein, HIST1, PSM and RPL cluster genes, WNT, Rho GTPase signalling as well as chromatin organisation (Fig. 1f and Additional File 1: Fig.S4b).

Additionally, loss of H3K4me3 at genes of the HIST1 cluster was observed in GIC, a finding never previously reported in a glioblastoma context (Additional File 1: Fig.S4b and Additional File 3: table S1), and of potential interest given that epigenetic downregulation of the *HIST1* locus has been linked to better prognosis in a proportion of acute myeloid leukaemia patients [27]. We found loss of H3K27ac at genes related to Proteasome subunits type A, B, C and D (*PSM*), in particular *PSMD1* and *PSMD3* (Additional File 1: Fig.S4b and Additional File 3: table S1), which have been shown to act as tumour suppressors and inhibit Wnt signalling [28] in other cancers, though not yet described in glioblastoma. Finally, loss of H3K27ac is found in genes related to cellular response to stress, supporting the notion that cancer cells may not activate cell cycle arrest and/or apoptosis as effectively as non-neoplastic cells in a stress response context.

In summary, we show redistribution of functionally critical HM across the genome in neoplastic stem cells which affects biological processes known to play a role in glioblastoma pathogenesis, raising the possibility that epigenetic remodelling contributes to their deregulation. The approach also identifies chromatin remodelling at genes/pathways not yet linked to glioblastoma.

### Integrative analysis of single histone modifications and transcriptome reveals a direct impact on gene expression and identifies activation of a gastrulation differentiation programme in GIC

**Fig. 2:**
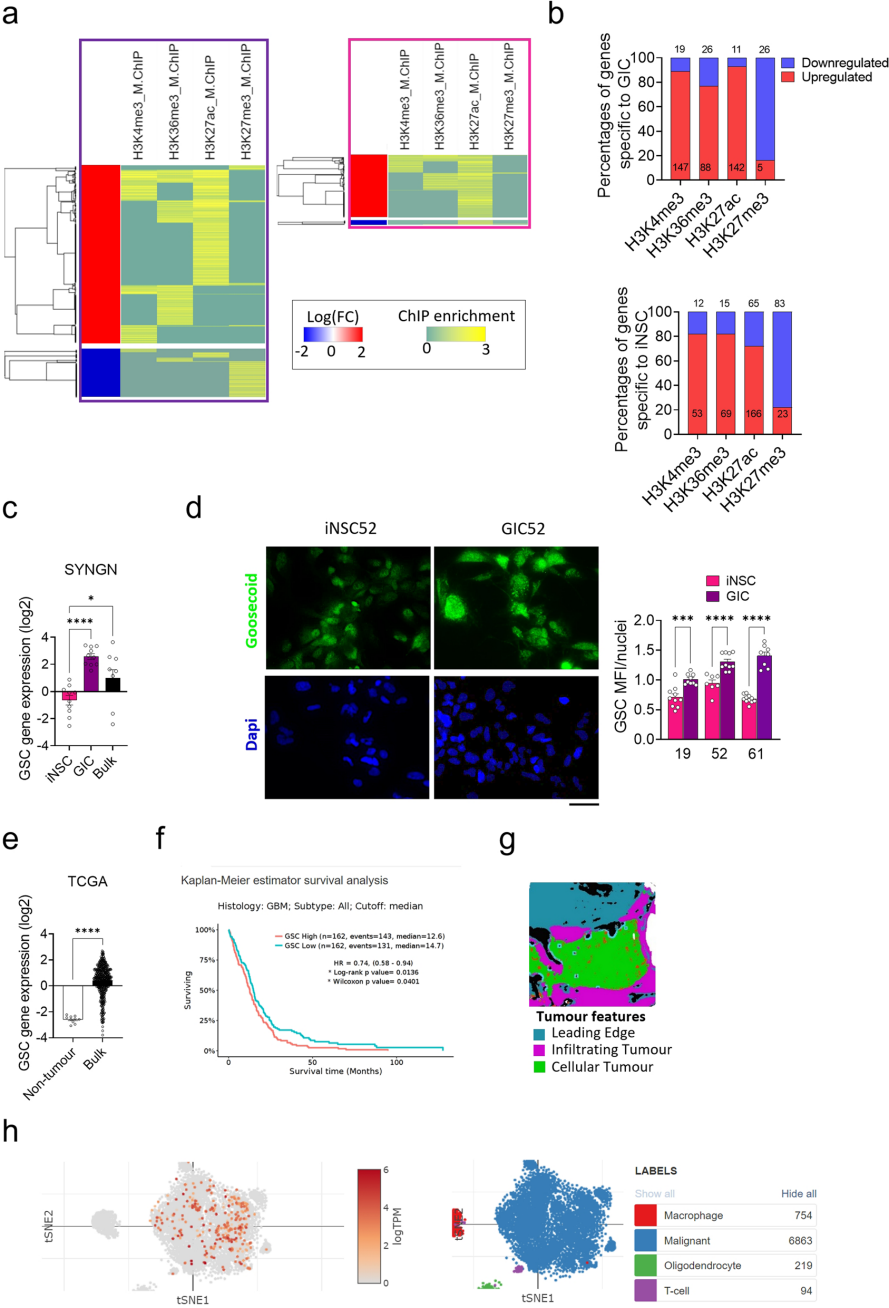
Integrative analysis of single histone modifications and gene expression reveals dynamic and synergistic epigenetic regulation of pathways involved in glioblastoma pathogenesis. **a** Pearson correlation heatmaps of integrative analysis of ChIP Seq data for each HM and RNAseq dataset [23] for genes only found in GIC (left) or iNSC (right) for at least one HM. RNAseq data are represented as log fold change of Differentially Expressed (DE) genes between GIC and iNSC: logFC DE > 1 and < − 1 when genes are up (red section) and downregulated (blue section) in GIC as compared to iNSC respectively (left). LogFC DE > 1 and < − 1 when genes are down and upregulated in iNSC as compared to GIC respectively (right). **b** Percentages of upregulated (red) and downregulated (blue) genes in iNSC as compared to GIC (top) and in GIC as compared to iNSC (bottom) for each HM based on transcriptomic dataset from the SYNGN cohort [23]. Number of genes is also specified for each condition. **c** mRNA expression of *GSC* in iNSC, GIC and bulk tumour from the RNAseq dataset of the SYNGN cohort [23] (left) and in bulk tumour and non-tumour samples from TCGA dataset [29]. Results are expressed in log 2 (tpm) transcript per million (tpm). One-way ANOVA test. *p value < 0.05, **p value < 0.01 and ***p value < 0.001. **d** Representative immunofluorescent images for GSC (green) in iNSC and GIC from patient 52. Nuclei are counterstained with DAPI. Scale bar: 50 µm. Quantification is shown as Mean Fluorescence Intensity (MFI) standardised by the number of nuclei. One-way ANOVA test. *p value < 0.05, **p value < 0.01, ***p value < 0.001, ****p value < 0.0001. **e** *GSC* gene expression in non-tumour and bulk primary glioblastoma tumour (left panel). t-test. *p value < 0.05, **p value < 0.01 and ***p value < 0.001. **f** Survival curve of glioblastoma patients with high and low expression of *GSC* gene (right panel). Source: TCGA [29] Stat test: log-rank, * *p* value < 0.05, ** *p* value < 0.01 and *** *p* value < 0.001. **g** Spatial expression of *GSC* in glioblastoma bulk samples, analysed on Ivy –GAP [30]. The left panel shows an example of histological anatomic structure identified in a sub-block and the right panel represents the expression of *GSC* in RNAseq data from anatomic structures shown as log2 normalised gene expression. Leading Edge defined as the border of the tumour, where ratio of tumour to normal cells is 1–3 / 100. Infiltrating tumour defined as the intermediate zone between leading edge and cellular tumour, where ratio of tumour to normal cells is 10–20 /100. Cellular tumour defined as tumour core, where tumour to normal cells is 100–500 / 1. One-way ANOVA test. * *p* value < 0.05, ** *p* value < 0.01 and *** *p* value < 0.001. **h** Single-cell RNAseq data showing *GSC* expression (left panel) in scRNAseq of glioblastoma samples in clusters defined in [31] (right panel). Data are plotted as tSNE, with logTPM expression ranging from light orange to dark

To begin understanding the functional impact of the chromatin remodelling identified in GIC, we integrated transcriptomic data [23] with the ChIP Seq datasets (Additional File 1: Fig.S5a). As expected, active regulatory regions defined by H3K4me3, H3K36me3 or H3K27ac peaks are found at upregulated genes whilst H3K27me is found mostly at downregulated genes in GIC as well as in iNSC, although here less prominently (Additional File 1: Fig.S5a-c). However, active and repressive marks are also found at downregulated and upregulated genes respectively, raising the possibility that the expression of these genes is regulated by alternative/additional mechanisms (Additional File 1: Fig.S5a). Interestingly, when the analysis was focused on the regions differentially bound uniquely in GIC or iNSC (Fig. 2a and Additional File 1: Fig.S5d), an enrichment of genes with an expression concordant with the HM was observed (Fig. 2b). Over 90% of genes displaying a gain of activating HM (H3K4me3, H3K36me3 and H3K27ac) were upregulated in GIC as compared to iNSC, whilst 80% of genes gaining H3K27me3 were downregulated (Fig. 2b top), with a similar observation also made for iNSC (Fig. 2b bottom), hence suggesting that the redistribution of the HM observed is likely to have a functional impact.

To identify pathways directly regulated by HM in GIC, we selected the concordant genes (defined as those that show a relationship between histone modifications and gene expression levels consistent with our current understanding of the biological read-out of that regulation, i.e. activating HM/upregulation of expression and repressive HM/downregulation of expression) for each HM and performed GSEA (Additional File 1: Fig.S5e and b, Additional File 3: table S2). In the first instance, we confirmed the results of the SHM analysis that pathways, described in previous studies as deregulated in glioblastoma pathogenesis, are epigenetically regulated, including pathways related to neoplastic transformation (Receptor Tyrosin Kinase (RTK) signalling (EBRR2) [32], WNT signalling [33], GPCR signalling [34]), neuronal systems (potassium channels [35]), cellular response to stress and ALK signalling [36, 37], as well as genes involved in circadian clock regulation [36, 38, 39] (Additional File 1: Fig.S5e and Additional File 3: table S2). Pathways involved in modulation of the inflammatory microenvironment via interferon signalling (antigen presentation, cytokine signalling and interferon signalling) [40] were also identified, in keeping with an intrinsic regulation of the inflammasome mediated by the redistribution of HM in tumour cells (Additional File 1: Fig.S5e and Additional File 3: table S2). Interestingly, we found decreased expression of a group of 37 ribosomal protein coding genes associated with the combined loss of the three activating histone modifications (Additional File 1: Fig.S6a and Additional File 3: table S2), in keeping with chromatin remodelling impacting ribosome biogenesis [41].

Importantly, our analysis also identified novel pathways/genes specifically deregulated in GIC as compared to iNSC including pathways associated with activation of GABA B receptors signalling (GABA B receptor activation and activation of GABA B receptor) and activation of the gastrulation pathway (Additional File 3: table S2) mediated by the gain of H3K4me3 and upregulation of Goosecoid (GSC) (Additional File 1: Fig.S6b). We confirmed *GSC* upregulation in GIC as well as in the bulk tumour as compared to iNSC by qPCR (Fig. 2c) and at the protein level (Fig. 2d and Additional File 1: S6c and d). Leveraging the Cancer Genome Atlas Program (TCGA) [29], we confirmed its upregulation in additional glioblastoma samples as compared to non-tumour tissue (Fig. 2e) and demonstrated a link to poorer prognosis in higher expressors in this cohort of patients (Fig. 2f). At regional level (Glioblastoma Atlas Project—IvyGap) [30], *GSC* expression is highest in the cellular tumour (tumour core) and show intermediate expression in the infiltrating tumour (ratio of tumour-to-normal cells 10–20/100) as compared to leading edge (ratio of tumour-to-normal cells 1–3/100 (Fig. 2g), with *GSC* being exclusively expressed in malignant cells at single-cell transcriptomic level (Single Cell Portal, The Broad Institute) [42] (Fig. 2h). A link between the expression of *GSC* and the type of cellular differentiation was not observed in GBMap [43] (Additional File 1: Fig.S6e).

In summary, we show that the redistribution of key regulatory HM in the neoplastic stem cell context has an impact on gene expression and identify novel epigenetic regulatory programmes, including reactivation of a gastrulation differentiation programme in glioblastoma.

### Chromatin states segmentation confirms the contribution of chromatin remodelling to regulation of mechanisms of glioblastoma pathogenesis

**Fig. 3:**
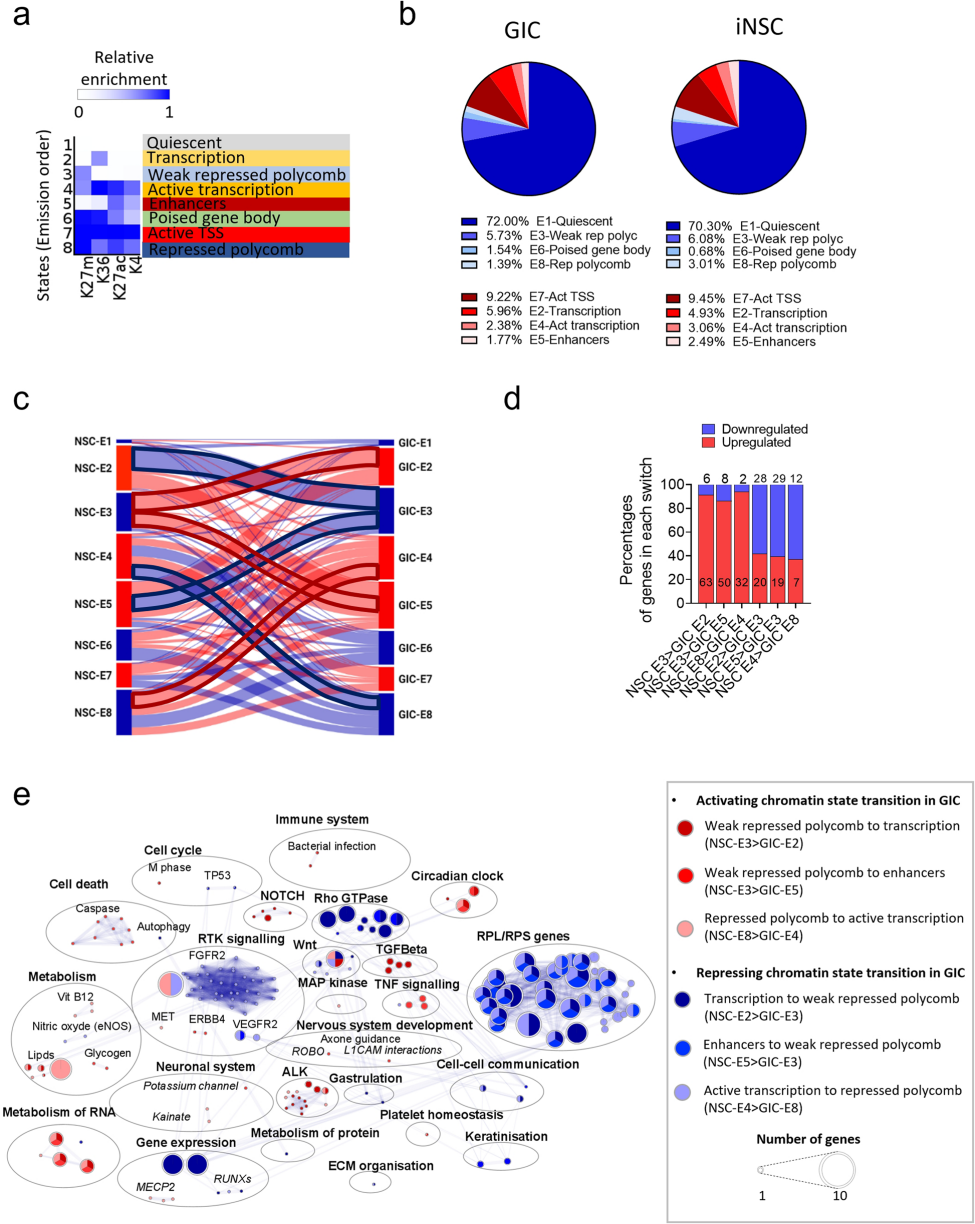
Comparative analysis of the functional impact of chromatin states dynamics in GIC and iNSC using automatic fragmentation analysis. **a** Chromatin states defined by enrichment of HM using ChromHMM [44]. Probabilities of each HM in chromatin states are depicted as a heatmap. **b** Pie charts show percentages of peaks in each chromatin state in GIC (left) and iNSC (right). **c** Sankey diagram shows the switch of peaks from one chromatin state in iNSC to another in GIC. The thickness of the links is proportional to the number of peaks included. Flows with the highest number of peaks between two opposite state functions are highlighted in bold red (activating transition in GIC) and blue (repressing transition in GIC). **d** Percentages of upregulated (red) and downregulated (blue) genes in the chromatin states of interest based on transcriptomic dataset from the SYNGN Cohort [23]. Number of genes is also specified for each condition. **e** Visualisation of the enriched pathways identified in GIC from genes activated in GIC as compared to iNSC and from genes inactivated in GIC as compared to iNSC. Pathways are annotated based on pathways enrichment analysis performed with Reactome and represented as circle, colours represent each histone (see legend), size of the circle is proportional to the number of genes involved in the pathway (FDR < 0.05)

Next, we set out to capture the complexity of the epigenetic deregulation in glioblastoma in a systematic manner. Using ChromHMM genomic segmentation [44], we explored the unsupervised combinatorial patterns of the four histone marks in an 8-state model and predicted active and inactive chromatin states at specific genomic features in GIC as compared to iNSC (Fig. 3a). The 8 chromatin states were defined as active states—“transcription”, “active transcription”, “enhancers” and “active TSS”—as well as inactive states—“quiescent”, “weak repressed polycomb”, “repressed polycomb” and “poised gene body”. We first trained the model on iNSC and applied it to GIC and vice versa (Additional File 1: Fig.S7a) and given that a similar level of generalisation was observed (Additional File 1: Fig.S7a), we selected the iNSC model for the downstream analysis. Assessment of percentages of the genome in each state showed that the majority was in weak transcription/quiescent state (72 and 70.3%) and 1–8% in an enhancer state (Fig. 3b and Additional File 1: Fig.S7b) in both GIC and iNSC, similar to previous work [45]. Noticeably though, the distribution of the peaks across the states is different in iNSC and GIC with a higher and lower respective percentage of peaks in poised gene body state (1.54 vs 0.68%) and in repressed polycomb state (1.39 vs 3.01%) in GIC as compared to iNSC (Fig. 3b). In the activating states, we find more peaks in the transcription state in GIC than in iNSC (5.96 vs 4.93%) whilst fewer peaks are to be found in the enhancer state (1.77 vs 2.49%) (Fig. 3b), indicating a loss of enhancer activity in GIC. The genomic region annotation of the peaks for each state did not reveal striking qualitative differences between GIC and iNSC (Additional File 1: Fig.S7c) and the comparison of peaks in each chromatin state revealed that regions are common between GIC and iNSC highlighting the similarities between the two cell types (Additional File 1: Fig.S7d).

As for the SHM analysis, we focused on the regions uniquely found in GIC and iNSC (Additional File 1: Fig.S7d) and integrated these results with the transcriptomic data to focus on functionally relevant events. We show 87, 85, 78 and 63% of concordance between active chromatin states (respectively transcription, active transcription, enhancers and active TSS) and gene upregulation both in GIC and iNSC (Additional File 1: Fig.S8a left) thus confirming a strong epigenetic regulation of gene expression in this cell type. However, lower percentages of downregulated genes, 63, 75 and 59%, are found in repressing chromatin states (respectively quiescent, weak repressed polycomb, poised gene body and repressed polycomb) (Additional File 1: Fig.S7a right) in both cell types, in keeping with the more nuanced impact on transcription regulation of these chromatin states, which may lead to low reduction in gene expression which may not have been captured by the thresholds we used for DE analysis.

GSEA of concordant genes confirmed epigenetic regulation of pathways known to be contributing to glioblastoma pathogenesis such as transcription factors *RUNX* [46], *SMAD*s [21, 47] and PPARA [48], neddylation of protein [49], homeostasis disorder including Basigin [50] and Kallikrein/kinin complex [51]. Conversely, loss of repressing states in GIC leading to upregulation of genes involved in extra-cellular matrix organisation pathways (Laminin interactions, integrin cell surface interactions and ECM proteoglycans) and necrosis was observed (Additional File 1: Fig.S8b). Enrichment of inflammasome pathways (interferon signalling, antigen presentation and cytokines signalling) and circadian clock-related pathways with gain of activating chromatin states and loss of repressing states respectively confirmed our previous finding (Additional File 1: Fig.S8b and Additional File 3: table S3). Within the inflammasome, the interferon-related pathways are consistently enriched because of the upregulation of two of the 2’−5’-oligoadenylate synthases family (*OAS1* and *OAS3*). *OAS1* is known to be upregulated [52] and hypomethylated [53] in glioblastoma and its silencing leads to an increase of temozolomide-sensitivity in vitro [52]. Interestingly, we find an upregulation of *OAS1* and* 3* in our dataset in GIC as compared to iNSC (Additional File 1: Fig.S8c), that is linked to worse prognosis for *OAS3*, as indicated by poorer patient survival (TCGA database) [29] (Additional File 1: Fig.S8d). Regional expression dataset (IvyGap) [30] revealed no difference between the locations analysed (leading edge, infiltrating tumour and cellular tumour) for OAS1 with the expression not being tumour cell-specific, whilst OAS3 is significantly more expressed in the cellular tumour (tumour core) (Additional File 1: Fig.S8e). Non-exclusive expression in the tumour cells, with immune cells including macrophages and T cell and oligodendrocytes also expressing these genes was confirmed at single-cell transcriptomic level (Additional File 1: Fig.S8f) in keeping with their immune regulatory role.

Similar to our findings in the SHM analysis, epigenetic-mediated downregulation of *RPL* and *RPS* genes is confirmed also with the ChromHMM approach, mostly mediated by the loss of the 4 activating chromatin states and the gain of poised gene body state (Additional File 1: Fig.S9a and Additional File 3: table S3). Signalling pathways related to the receptor tyrosine kinase MET [54] and ECM organisation [55] are also found enriched by genes epigenetically downregulated in GIC as compared to iNSC (Additional File 1: Fig.S9a) as well as deregulation of keratinisation, previously described in a glioblastoma context [56]. Importantly, we identified an enrichment for cellular response to hypoxia (Additional File 1: Fig.S9a), potentially mediated by the epigenetic downregulation of the Ubiquitin-conjugating enzyme E2 D1 (*UBE2D1*) known to be responsible for the ubiquitination of hypoxia-inducible transcription factor HIF-1 alpha leading to its degradation. Whilst it has not been described in glioblastoma to date, our data suggests that in GIC, *UBE2D1* is in a poised state which could allow the cancer cells to respond quicker to cellular stress such as hypoxia by decreasing HIF1-apha degradation and promoting angiogenesis and hence tumour maintenance in a hypoxic environment. Interestingly, its low expression is linked to poor prognosis (Additional File 1: Fig.S9b) and regional transcriptomic shows it to be expressed by malignant cells as well as other brain and immune cells (Additional File 1: Fig.S9c). Interestingly, integrating proximal enhancer regions uniquely found in GIC or iNSC with the RNAseq data shows that genes regulated by GIC only enhancers, mainly involved in glial cell differentiation pathways (Additional File 1: Fig.S9e, left), are significantly more likely to be upregulated in GIC (Additional File 1: Fig.S9d). Conversely, genes regulated by NSC only enhancers, regulating cGMP-mediated signalling (Additional File 1: Fig.S9e, right), are significantly more likely to be downregulated in GIC (Additional File 1: Fig.S9d). This supports a direct impact of the identified epigenetic switches in GIC on gene expression. Note that our results showed that only approximately 5% of changes in enhancers can be linked with differential expression of genes; a finding which is expected since we considered only proximal enhancers, enhancers often display redundancy [57] and the thresholds used in this type of analysis might not capture smaller but significant changes in gene expression.

In summary, unbiased genomic segmentation confirmed that chromatin states remodelling contributes to regulation of key oncogenic pathways in glioblastoma.

### Analysis of transitioning peaks from a repressing state in iNSC to an activating state in GIC and vice versa identifies GABBR2 and SMOX as novel druggable target genes involved in migration and invasion of tumour cells

To capture the potentially more functionally relevant chromatin conformation changes and to capitalise on the patient-specific comparison enabled by the SYNGN platform, we then focused on peaks that were transitioning/switching from one state in iNSC to another state in GIC in at least two patients of the cohort (Fig. 3c and Additional File 1: Fig.S10a). We observed switching in all 8 chromatin states with most switches found in state 4 (active transcription) in both iNSC and GIC and less switches in state 6 (weak transcription/quiescent) (Fig. 3c and S10a) with more peaks observed in repressing switches as compared to activating ones (57% vs 43%) (Additional File 1: Fig.S10b). Notably, the most frequent switches occurred within weak polycomb, transcription, active transcription, and enhancers (Fig. 3c and S10a). Genome annotation of the peaks revealed that they are mostly found in promoter regions for the activating switches “weak polycomb to enhancers”, “repressed polycomb to active transcription” and repressing switches “transcription to weak repressed polycomb”, “enhancers to weak repressed polycomb”; whilst most peaks are in the intron regions for the activating “weak repressed polycomb to transcription” and the repressing “active transcription to repressed polycomb” switches (Additional File 1: Fig.S10c). Notably, the integration with transcriptomic dataset consistently showed that the predicted functional impact of activating switches is higher than the repressing switches (over 90% concordance vs over 60%) (Fig. 3d).

The 6 switches with more peaks transitioning between states were selected for further analysis, including 3 switches from an inactivating state in iNSC to an activating state in GIC (weak polycomb to transcription; weak polycomb to enhancers and repressed polycomb to active transcription) and 3 switches from an activating state in iNSC to an inactive state in GIC (transcription to weak repressed polycomb, enhancers to weak repressed polycomb and active transcription to repressed polycomb) (Fig. 3c and Additional File 1: Fig.S10a). GSEA analysis of the differentially regulated genes confirmed as significantly enriched (FDR < 0.05) several pathways identified in the SHM analysis including RTK and WNT signalling, *RPL/RPL* genes, which are transitioning from activating chromatin states in iNSC (“transcription” or “enhancers”) into a repressing state in GIC (“(weak) repressed polycomb”) as are the circadian clock-related pathways (Fig. 3e and table S4). It has been previously described that the downregulation of two key transcription factors BMAL1 or CLOCK in GIC induces cell cycle arrest and apoptosis [36]. Heterodimer of CLOCK and BALM1 are a major transcriptional regulator of the circadian clock mechanism in mammals. Our analysis reveals an epigenetically mediated upregulation of the Neuronal PAS domain protein 2 (*NPAS2*), which is a paralog of *CLOCK*, able to functionally substitute for it in the regulation of circadian rhythmicity [58].

**Fig. 4:**
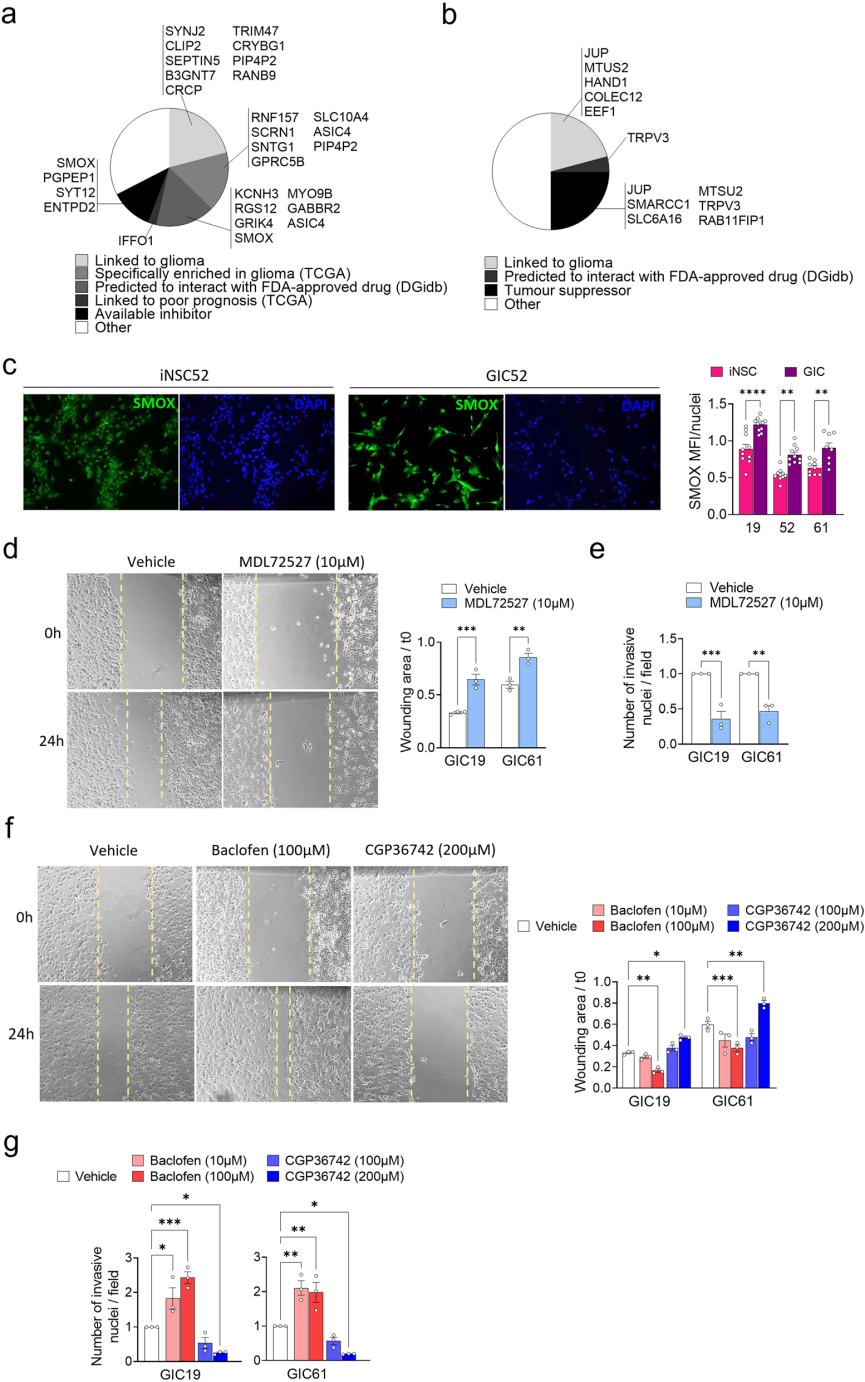
SMOX and GABBR2 are regulated by transitioning peaks in GIC. **a** Venn diagrams show the epigenetically upregulated genes newly identified in GIC classified in subgroups taking into account feasibility of molecular validation or suitability for clinical application. **b** Venn diagrams show the epigenetically downregulated genes newly identified in GIC classified in subgroups taking into account feasibility of molecular validation or suitability for clinical application. **c** Representative images of SMOX protein expression (green) in iNSC and GIC from patient 52. Nuclei are counterstained with DAPI. Scale bar: 100 µm. Quantification of results is shown as Mean Fluorescence Intensity (MFI) standardised by the number of nuclei for three patients. One-way ANOVA test. *p value < 0.05, **p value < 0.01, ***p value < 0.001, ****p value < 0.0001. **d** Representative bright-field images show wound healing assay in GIC19 cells treated for 24 h with 10 µM of SMOX inhibitor MD72527. Quantification of results is expressed in wound area at 24 h standardised on t0 for GIC from two patients. Experiments have been performed three times. One-way ANOVA test. *p value < 0.05, **p value < 0.01, ***p value < 0.001. **e** Invasion assay quantification of results expressed in fold change of the average number of nuclei per image field of GIC from patients 19 and 61 treated with 10 µM of SMOX inhibitor MDL72527 standardised on the vehicle. Experiments have been performed three times. One-way ANOVA test. *p value < 0.05, **p value < 0.01, ***p value < 0.001. **f** Representative bright-field images show wound healing assay in GIC19 cells treated for 24 h with 100 µM of GABBR2 agonist Baclofen or 200 µM of GABBR2 antagonist CGP36742. Quantification of results is expressed in wound area at 24 h standardised on t0 for GIC from two patients. Experiments have been repeated three independent times. One-way ANOVA test. *p value < 0.05, **p value < 0.01, ***p value < 0.001. **g** Invasion assay quantification of results expressed in fold change of the average number of nuclei per image field of GIC from patients 19 and 61 treated with 100 µM of GABBR2 agonist Baclofen or 200 µM of GABBR2 antagonist CGP36742 standardised on the vehicle. Experiments have been repeated three independent times. One-way ANOVA test. *p value < 0.05, **p value < 0.01, ***p value < 0.001

Next, we reviewed the existing literature on the genes affected by these chromatin state transitions and identified 42/102 upregulated genes and 27/50 downregulated genes as never previously described in a glioblastoma context (Additional File 3: table S5). We focused our attention on the activated genes as they could be potentially more easily targetable pharmacologically, among these 9 had been linked to low grade glioma, 7 are found to be specifically enriched in glioma among other cancers (TCGA) [29], 7 are predicted to interact with an FDA-approved drug (DGidb query [59]), one is linked to poor prognosis (TCGA) [29] and 5 can be inhibited by a commercially available small molecule (Fig. 4a).

Spermine oxidase (SMOX) catalyses the oxidation of spermine to spermidine and is found to transition from repressed polycomb to transcription through the gain of H3K27ac and H3K36me3 (Additional File 1: Fig.S11a). Spermidine is a polyamine, known to be increased in brain tumours including glioblastoma and medulloblastoma [60], although the role of SMOX in this process has not been characterised. *SMOX* expression is not linked to poor prognosis (Additional File 1: Fig.S11b), but regional and single-cell transcriptomic reveal that it is highly expressed in the tumour core as well as in peripheral areas where it is mostly expressed by malignant cells (Additional File 1: Fig.S11c and d). Leveraging the GBmap dataset [43], we show that *SMOX* is preferentially expressed in astrocytes (Additional File 1: Fig.S11f), raising the possibility that its expression could be linked to lineage specification in glioblastoma. Gene expression analysis by qPCR (Additional File 1: Fig.S11f) and by immunostaining (Fig. 4c and Additional File 1: Fig.S11g) confirmed higher expression of *SMOX* in GIC as compared to iNSC in three patients of the SYNGN cohort. We next aimed to modulate *SMOX* activity via a pharmacological approach with its inhibitor MDL72527 which is able to penetrate the blood brain barrier and has been shown to exert a cytotoxic effect on colon carcinoma cell lines ^57^. MDL 72527 is a selective and irreversible inhibitor of polyamine oxidases, particularly spermine oxidase (SMOX), which has shown to have a neuroprotective effect in models of neurodegenerative diseases and ischemic brain injury [61]. We show that SMOX inhibition does not affect the proliferation of GIC or iNSC (Additional File 1: Fig.S11f); however, it promotes tumour cell migration, as assessed by wound healing assay, including assessment of gap closure (Fig. 4d) and invasion (Fig. 4e and Additional File 1: Fig.S11g).

*GABBR2* has been consistently identified in all the analyses performed in our study and is found to transition from repressed polycomb to enhancers through the loss of H3K27me3 and the gain of H3K27ac (Additional File 1: Fig.S12a). We confirmed upregulation of its expression in GIC as compared to iNSC in three patients of the cohort (Additional File 1: Fig.S12b), with no effect on prognosis (Additional File 1: Fig.S12c) and regional and single cell transcriptomic analysis revealed that it is highly expressed in the tumour core as well as in peripheral areas where it is expressed by malignant cells as well as other brain and immune cells (Additional File 1: Fig.S12d,e). Leveraging the GBmap dataset [43], we show that *GABBR2* is preferentially expressed in neurons (Additional File 1: Fig.S12f), possibly implying a role in neuronal specification in glioblastoma. To explore a potential functional impact of GABBR2 modulation in GIC, we selected an agonist (BACLOFEN, a myorelaxant antispastic agent) and an antagonist (CGP36742, selective and potent compound able to penetrate the brain). Baclofen is a muscle relaxant and antispasmodic agent primarily used to treat muscle spasticity related to conditions such as multiple sclerosis and spinal cord injuries. It acts as an agonist at GABA B receptors in the central nervous system, which leads to reduced transmission of excitatory signals in the spinal cord and brain [62]. Although its direct role in glioblastoma therapy is not well established, its neuroprotective [63] and anti-inflammatory properties [64] suggest it may have therapeutic potential in modulating the tumour environment or managing symptoms associated with the disease. CGP36742 is an orally active, selective antagonist of the GABA_B receptor. It was developed as a research tool to study the role of GABA_B receptors in the central nervous system [65]. The compound inhibits GABA_B-mediated neurotransmission, which is implicated in various neurological processes, including learning and memory [65]. We show that pharmacological modulation of GABBR2 does not affect proliferation of tumour or non-neoplastic cells (Additional File 1: Fig.S12g) whilst its activation promotes migration (Fig. 4f top) and invasion of the tumour cells (Fig. 4g top and Additional File 1: Fig.S12h) in keeping with previous finding in breast cancer [66]. Importantly an opposite effect is observed when GIC cells are treated with the antagonist CGP36742, with inhibition of migration (Fig. 4f bottom) and invasion (Fig. 4g bottom).

Comparative analysis of switching chromatin states between GIC and iNSC identified novel pharmacologically targetable genes, including *SMOX* and *GABBR2* in a subgroup of glioblastoma, which could be further explored as new therapeutic approaches to counteract glioblastoma invasiveness.

## Discussion

Taking advantage of pairs of glioblastoma stem cells and their ontogenetically linked patient-matched neural stem cells, we identified significant chromatin remodelling in glioblastoma which is primarily driven by H3K27ac and H3K27me3, given the lower number of shared genes identified in these genomic regions between normal and neoplastic stem cells, as compared to regions decorated by H3K4me3 and H3K36me3 peaks. Overall, we found a strong concordance between HM/chromatin states and gene expression and globally we found a positive correlation between activating states and mRNA levels and anticorrelation between repressive states and mRNA levels, suggesting that our profiling captures the chromatin and transcriptional state of GIC. Complementary analytical approaches of this combination of HM integrated with transcriptome analysis defined functional regions of the GIC genome with increased or reduced expression of genes uniquely in GIC. Notably, in our datasets integration with the transcriptome consistently showed that the predicted functional impact of activating states/switches is higher than the repressing ones, suggesting that the chromatin state serves as a mechanism to maintain the constitutive high expression of genes important for neural stem cell identity and function. Redistribution of the Polycomb-mediated H3K27me3 mark was also observed, consistent with a model whereby GIC identity is in part due to H3K27me3-mediated silencing of genes although other mechanisms, such as for example H3K9me3, must contribute to downregulation of gene expression in these cells, particularly in a non-neoplastic context, given the lower concordant correlation with gene expression. Our findings support and further expand previous studies which have characterised the active enhancer landscape in GIC and primary glioblastoma tissue by means of integrative analysis of the HM H3K27ac and other (epi)genetic datasets with gene expression [21].

It is of interest that several of the molecular processes known to be involved in glioblastoma pathogenesis are either confirmed or identified as epigenetically regulated in this study. NOTCH 1 signalling has been previously described as activated in glioblastoma, including via epigenetic regulation mediated through H3K4me3 [67], and our data confirm this regulation hence also lending support to our experimental approach. We show loss of H3K36me3 and concomitant repression of the beta-catenin degradation pathway via Axin in keeping with existing knowledge of over activation of WNT pathway in glioblastoma [33]. Genes involved in tumour progression including non-integrin membrane-ECM interactions, *COL4A* and *PDGFA*, showed a gain of H3K4me3 in our dataset, in keeping with epigenetic regulation of these mechanisms being critical. GIC are equipped with a robust DNA repair mechanism mainly mediated by Homologous Recombination Repair (HRR) pathway [68], and we show its epigenetic regulation by gain of H3K36me3; as well as pathways involved in increased genomic instability through alteration of NHEJ/HDR pathways, which we show to be at least in part epigenetically regulated through the gain of H3K36me3. We identified an enrichment for cellular response to hypoxia, potentially mediated by the epigenetic downregulation of the Ubiquitin-conjugating enzyme E2 D1 (*UBE2D1*) known to be responsible for the ubiquitination of hypoxia-inducible transcription factor HIF-1 alpha leading to its degradation (provided by RefSeq, Mar 2011). Whilst it has not been described in glioblastoma to date, our data suggests that, in GIC, gene body region of *UBE2D1* is in a poised state which could allow the cancer cells to response quicker to cellular stress such as hypoxia by decreasing HIF1-apha degradation, promoting angiogenesis and tumour maintenance in a hypoxic environment. Interestingly, its low expression is linked to poor prognosis and spatial expression shows its expression by malignant cells as well as other brain and immune cells [31]. Our data suggest that the poor prognosis could be tumour-cell driven, given the epigenetic regulation observed in GIC; however, a contribution of the tumour microenvirorment cannot be excluded given the spatial expression pattern observed.

Interestingly, we also identified pathways regulated by a combination of histone modifications, gain of H3K4me3, H3K36me3 and H3K27ac or loss of H3K27me3 respectively, including cellular response to stress and ALK signalling, for which a dysregulation has been described in glioblastoma [36, 37]. Pathways involved in the modulation of the inflammatory microenvironment via interferon signalling (antigen presentation, cytokine signalling and interferon signalling) which were shown to regulate cell death and mesenchymal phenotype [40] are epigenetically regulated in GIC, hence demonstrating a cell intrinsic regulation of the inflammasome mediated by the redistribution of HM in tumour cells. In 2019, Dong et al. showed that GIC can be targeted through the downregulation of the circadian clock genes *BMAL1* or *CLOCK* leading to cell-cycle arrest and apoptosis [36]. Moreover, CLOCK has been shown to promote tumour angiogenesis [39] and immunosuppression [38]. We show here that genes involved in circadian clock are epigenetically upregulated in a multi-factorial fashion by means of gain of the 3 activating HM included in our analysis (H3K4me3, H3K36me3 and H3K27ac).

Our study shows an epigenetic regulation of a large group of 57 ribosomal protein coding genes mediated by the loss of H2K27ac, 37 of which also showed decreased expression associated with the combined loss of the three activating HM. Dysregulation of ribosomal proteins (RPL/RPS) is a hallmark of cancer including glioblastoma [41, 69] and importantly ribosome biogenesis has been described to support the synthesis of protein involved in the differentiation process of NSC [70]. Downregulation of the expression of ribosomal genes contributes to myeloid lineage differentiation in bone-marrow–derived macrophages [71] and a recent study showed that GIC acquire an epigenetic immune editing process launching a myeloid-affiliated transcriptional programme as an immune evasion programme [72]; hence, it is possible to speculate that epigenetic regulations such as those described here could contribute to this plasticity.

Developmental programmes participating in tissue development and homeostasis re-emerge in tumours including glioblastoma, and reactivation of such aberrant expression programmes supports stemness, growth and migratory properties of the tumour cells [73]. We show loss of H3K27me3 and gain of H3K4me3 at the pluripotency markers, *NANOG*, *OCT3/4* and *SOX2*, in GIC, hence maintaining their embryonic-like gene expression signature, which contributes to drug resistance and recurrence [74]. Interestingly, we also identified loss of H3K27me3 in genes involved in pancreatic beta-cell development (*ONECUT3*, *ONECUT1*, *NKX6-1*, *NKX2-2*, *RFX6*, *MAML3*, *PTF1A*, *FOXA3*, *NEUROG3*) raising the possibility that the redistribution of H3K37me3 in GIC could lead to reactivation of the beta-cell transcription programme in these cells with potential impact on insulin production and glucose metabolism. This supports previous findings showing that activation of insulin-mediated signalling pathways in glioblastoma promotes proliferation and survival of the tumour cells through PI3K/Akt [75] and anti-glycemic therapy has been recently shown to enhance PI3K inhibitor efficacy in glioblastoma patients [76]. Finally, we identified the H3K4me3 mark and activation of the expression of Goosecoid (GSC) in GIC and demonstrated a link to poorer prognosis in higher expressors in the TCGA cohort of patients [29]. GSC is an homeobox gene expressed specifically in the dorsal blastopore lip of the gastrula which plays an important role in Spemann’s organiser formation. Its upregulation promotes tumour progression in several cancers, including breast, colorectal and hepatocellular carcinoma [77–79] by promoting invasion and migration, although the underlying mechanism is unclear. The activation of gastrulation-like programmes in glioblastoma further supports the notion that these tumours hijack developmental pathways to promote aggressive behaviours such as increased invasiveness and cellular plasticity. In the context of glioblastoma, our data raise the possibility that this mechanism is aberrantly re-activated through epigenetic deregulation and may contribute to tumour progression by enabling cancer stem cells to acquire mesenchymal characteristics, which are associated with enhanced migratory and invasive capabilities [64].

Epigenetic deregulation is an attractive albeit challenging therapeutic target for tumours, such as glioblastoma, where it is prominent. In fact, the only biomarker predicting response to a treatment which has some success in treating glioblastoma is methylation of the MGMT promoter predicting response to TMZ [5]. Our study has identified two novel druggable target genes, *SMOX* and *GABBR2*, which are differentially regulated and expressed in normal and neoplastic stem cells in glioblastoma. SMOX, a spermine oxidase, may influence tumour progression through the modulation of polyamine metabolism [80]. Increased SMOX activity leads to the degradation of spermine into spermidine and hydrogen peroxide, which increases oxidative stress and promotes DNA damage, cell proliferation and apoptosis resistance [80]. Polyamines, such as spermine, are critical for cell growth and differentiation and aberrant polyamine metabolism has been linked to cancer progression, including in glioblastoma [81]. Elevated levels of SMOX could, therefore, contribute to increased oxidative stress and altered cellular homeostasis, potentially promoting tumour growth and invasiveness. Conversely, its inhibition could decrease the tumour aggressiveness and improve response to therapy.

GABBR2 has been implicated in promoting cancer cell proliferation and invasion by interacting with other signalling pathways, such as the Wnt/β-catenin pathway [82]. This interaction can lead to enhanced tumour growth and resistance to apoptosis, contributing to the aggressive nature of glioblastoma. Modulation of GABBR2 could potentially affect tumour progression by altering these signalling pathways. Therefore, targeting GABBR2 could represent a promising therapeutic strategy to disrupt key pathways involved in glioblastoma progression. Further testing aiming at assessing the suitability of these genes as novel druggable target genes in glioblastoma is warranted.

## Conclusions

Chromatin remodelling in glioblastoma stem cells is different from the ontogenetically related neural stem cells. Chromatin state transition regulates key oncogenic pathways in glioblastoma. SMOX and GABBR2 are novel patient-specific epigenetically regulated potentially druggable targets in glioblastoma.

## Methods

### Human cell culture

Human primary GIC and iNSC cells originated from a novel experimental pipeline previously generated to derive cells from patients who underwent surgical resection of glioblastoma [23]. The use of human tissue samples was approved by the National Research Ethics Service (NRES), University College London Hospitals NRES Project ref 08/0077 (S Brandner); Amendment 1 17/10/2014. Briefly, GIC were isolated from fresh tumour tissue following a published protocol [83] and fibroblast from a small strips of dura mater. Fibroblasts were then reprogrammed into EPSCs [84], which were induced into iNSC using a commercially available kit following the manufacturer's protocol (Gibco, #A1647801).

GIC were cultured on laminin (Sigma, #L2020) coated tissue plates in NeuroCult NS-A proliferation kit media (Stem Cell Technologies Cat. #05751) supplemented with 1% penicillin/streptomycin solution (Sigma Cat. #P4458), heparin (2 µg/ml, Stem Cell Technologies Cat. #07980), mEGF (20 ng/ml, Peprotech Cat. #AF-315–09-1MG) and hFGF (10 ng/ml, Peprotech Cat. #AF-100-18B-50UG) and dissociated with accutase (Millipore, #SCR005) once they reached 70% confluence for replating. Cells were stored in liquid nitrogen in Stem Cell Banker freezing media (Ambsio ZENOAQ, #11890).

iNSC were cultured on geltrex (Gibco, #A1413302) coated tissue plates in Neural expansion media made of Neurobasal 0.5X (Gibco, # 21103049), Advanced™ DMEM⁄F-12 0.5X (Gibco, #12634010) supplemented with 1% penicillin/streptomycin solution and neural induction supplement (Gibco, #A1647801) and dissociated with accutase for replating. Cells were stored in liquid nitrogen in Synth-a-Freeze cryopreservation medium (Gibco, #A12542).

All cells were cultured at 37°C, 5% CO.

### RT qPCR

Total RNA was extracted from the cell pellet with RNeasy kit (Qiagen 74004) following the manufacturer’s protocol. 0.5 μg was retrotranscribed by SuperScriptIII (Invitrogen, 18080093). Five nanograms of cDNA template and SYBR Green primers was used to perform SYBR Green assay using SYBR Green PowerUp Master Mix (Applied Biosystems, A25742) and run on a StepOne Real-Time PCR System (ThermoFisher). The housekeeping genes GAPDH or ATP5B were used. GAPDH: FW 5′-CTGAGGCTCCCACCTTTCTC-3′; REV 5′- TTATGGGAAAGCCAGTCCCC-3′, ATP5B: FW 5′-GCGAGAAGATGACCCAGATC-3′; REV 5′-CCAGTGGTACGGCCAGAGG-3′, SMOX: FW 5′- TAACTCGTGACCTCCAGC-3′; REV 5′-GCGGCTAGCTCTACAGAA-3′, GABBR2: FW 5′- GACCATCTCAGGAAAGACTC-3′; REV 5′-GGTCTCGTTCATGGCATT-3′.

### Immunofluorescence

Adherent cultures of GIC and iNSC on a glass coverslip were washed once with PBS then fixed with PFA4% for 30 min at room temperature. After three washes with PBS, blocking with 10% goat normal serum, 0.1% Triton X100 PBS for 30 min at room temperature was performed prior to incubation with primary antibodies: 1/100 anti-SMOX (Thermo Fisher, PA5-100112), anti-GSC (Thermo Fisher, MA5-38019) at 4° overnight. After three washes with PBS and 1 h incubation with 1/200 secondary antibodies diluted in PBS, cells were washed again with PBS, and coverslip were mounted on SUPERFROST slides with mounting media containing DAPI for nuclear counterstaining (ProLong™ Gold Antifade Mountant, Invitrogen P36930). Microscope analysis was performed with Leica DM5000 EpiFluorescence.

### Drug treatment

In vitro drug treatments in adherent cells were performed on 5k cells plated in 96-well plates coated with geltrex and laminin for iNSC and GIC culture respectively. A 3-day treatment was performed with increasing doses of GABBR2 agonist CGP36742 (MedChem Express HY-121599 at 100 and 200 μM), GABBR2 antagonist Baclofen (Tocris, 0417 at 10 and 100 μM) and SMOX inhibitor MDL72527 (Sigma, M2949 at 10 μM) or vehicle. At end-point, cell viability and cytotoxicity were measured with CellTiter-Glo Luminescent Cell Viability Assay with CellTox Green Cytotoxicity Assay (Promega kits G7570 and G8741).

### Wound healing migration assay

5 × 10^4^ cells of GIC were seeded per well in a 96-well plate coated with laminin (Sigma #L2020). The next day, a vertical scratch was made with a 10-μl tip and 50 µg/mL mitomycin C (Sigma Aldrich) was added to the growth medium to inhibit the cell proliferation. Picture at × 10 magnification was taken following the scratch, as D0, and location of the picture was marked for each well on the plate’s lid. Pictures were taken at marked location for each well after 24 h incubation. Surface area of the wound was calculated with ImageJ. Experiments were performed 3 times with 2 technical replicas for each time.

### Invasion assay

Transwell inserts with 8.0-µm pores (Sarstedt Cat. #89.3932.800) were placed into wells of a 24-well plate and coated with 100 µL of GelTrex. A total of 100,000 GIC cells were then seeded into the transwell insert in 200-µL media with additional 700 µL of normal growth media was added to the bottom chamber of the well. Cells were then incubated in normal growth conditions for 24 h, at which point cells on the inside of the transwell were removed using a cotton bud dampened with DPBS. Once cells inside the transwell were removed, cells on the bottom of the transwell were fixed using methanol, pre-chilled at − 20 °C, for 5 min at room temperature. After fixation, the bottom of the transwell was washed twice for 5 min using DPBS. The membrane of the transwell was then cut out and mounted onto a microscopy slide with mounting media including DAPI (ProLong™ Gold Antifade Mountant, Invitrogen P36930). Transwell membranes were then analysed at the microscope and five representative images of nuclei on each membrane captured. Each experiment was repeated three times at different passages for each cell line. Each time, two technical replicate membranes were imaged. Finally, the number of nuclei in each image field was counted, using ImageJ software, to ascertain how many cells migrated across the membrane.

### RNA extraction, RT and qPCR

Total RNA was isolated from cell pellets with RNeasy Micro purification kit (Qiagen, 74104) and digested with DNaseI (Applied Biosystems). The cDNA synthesis was carried out with SuperScript III Reverse Transcriptase Kit (Invitrogen) following the manufacturer’s protocol. Analysis of gene expression was performed with the Applied Biosystems 7500 Real-Time PCR System using SYBR Green PCR Master Mix (Applied Biosystems) according to standard protocols. Technical triplicates for each sample were analysed. The Ct values of all the genes analysed were normalised to the average Ct of ACTB, and *ATP5F1B* and 2ΔCts were calculated on iNSC value. Primers used in SYBR Green qPCR are the following. *ACTB*: FW 5′-GCGAGAAGATGACCCAGATC-3′, REV 5′-CCAGTGGTACGGCCAGAGG-3′; *ATP5F1B*: FW 5′-CCCAGGCTGGTTCAGAGGT-3′, REV 5′-AGGGGCAGGGTCAGTCAAG-3′; *SMOX*: FW 5′-TAACTCGTGACCTCCAGC-3′, REV 5′-GCGGCTAGCTCTACAGAA-3′ and *GABBR2* FW 5′-GACCATCTCAGGAAAGACTC −3′, REV 5′-GGTCTCGTTCATGGCATT-3′.

### Statistical analysis for the wet lab experiments

Sample processing was carried out blinded. Statistical analysis was performed using GraphPad software unless otherwise stated. Significance was determined with *t*-test, one-way ANOVA (with Sidak’s test), or two-way ANOVA as appropriate, and displayed as the mean ± standard error (SEM). *p* < 0.05 was considered significant. Significance was indicated with asterisks: *p < 0.05; **p < 0.01; ***p < 0.001. Outliers were considered those data points furthest from the median value.

### Chromatin immunoprecipitation (ChIP) assays

H3K4me3, H3K36me3, H3K27ac and H3K27me3 ChIPs were performed using ChIP-IT High Sensitivity kit (Active Motif) following the manufacturer’s instructions. Briefly, 6 × 10^6^ cells were fixed with the formaldehyde-based fixing solution for 15 min at room temperature and lysed with provided lysis solution supplemented with protease inhibitors. Next, nuclei pellets were lysed, and chromatin sonicated with Bioruptor Plus sonication device (Diagenode) to obtain DNA fragments within the recommended 200–1200-bp range. In total, 25 µg of sheared chromatin was then incubated with 4 µg of antibody against H3K4me3 (Diagenode, C15410003-50), H3K36me3 (Abcam, ab9050), H3K27ac (Abcam, ab4729) or H3K27me3 (Diagenode C15410195) overnight at 4 °C with rotation. Following incubation with Protein G agarose beads, bound chromatin was washed, eluted and purified following the manufacturer’s protocols. Validation by qPCR-ChIP on target genes was done before proceeding to sequencing. ChIPed DNA was end-repaired, A-tailed and adapter-ligated before size selection and amplification. The obtained libraries were QC’ed and multiplexed before 75-bp paired-end sequencing on HiSeq4000 (Illumina).

### Computational analysis of ChIP Seq datasets

The quality of ChIP Seq samples was first assessed via FastQC and TrimGalore, removing low-quality and adapter sequences. The average Phred score of the surviving reads across all samples was 30 and the average sequencing depth was 36.3 M (min = 22.1 M, max = 56.7 M). The alignment to the Ensembl GRCh38 human reference genome was performed via Bowtie v2.3.4 [85] with default parameters, in concomitance with the usage of samtools [86] for the post processing and sorting of the Binary Alignment Map (bam) files. Exploratory tools such as deeptools [87], plotCorrelation, plotPCA and plotFingerprint on Python v2.7.15 were used to further assess sample characteristics and to address the potential presence of outliers. After performing post-alignment quality checks, peaks were called via the MACS2 algorithm [88] using the corresponding input background. The shifting model was disabled to make different datasets comparable and the “–broad” option was enabled for the analysis of the histone mark H3K27me3. A minimum fold enrichment of 2 was selected with an FDR of 0.05, in both narrow peak and broad peak (–broad-cut-off) statistical analyses. The Bioconductor packages in R GenomicRanges [89] and ChIPSeeker [90] were used to find regions of consensus peaks between the two cell lines, for each antibody/condition, and to annotate them based on the location with respect to the nearest transcription start site (TSS): promoters (within 3 kb from the TSS), exons, introns, 5′ UTR, 3′ UTR and distal intergenic. The latter was merged with so-called downstream regions. The versions of all relevant Bioconductor packages were compatible with R v3.5.3.

### ChIP Seq heatmap generation

The deeptools algorithms [91] bamCompare, bigWigCompare and plotHeatmap were used to produce relevant bigwig files and heatmaps, to assess region-wide and genome-wide coverage. Specifically, coverage tracks were first obtained by normalising each sample alignment file against the corresponding input (–operation log2ratio) and then pooled at both replicate and cell line levels (–operation mean), to obtain a representative sample for each antibody/condition. The regions chosen to be visualised in the heatmaps were those of consensus peaks (details in the figure legends), centred on their nearest TSS.

### Differential analysis for ChIP Seq data

The differentially bound sites (DBS) comparing GIC to iNSC were found using the R package DiffBind with default parameters. Finding regions with different functionality by identifying the various combinations of histone markers (e.g. H3K27ac lacking H3K4me3 for potential enhancer) were conducted using HOMER v4.11. Finally, R packages ChIPpeakAnno and Hsapiens.UCSC.hg38 were used to annotate the functional regions. The annotated peak results were compared to the list of differentially expressed genes from the RNA-seq ^1^. A Differentially Expressed (DE)/Differentially Bound Site (DBS) pair is considered if the gene regulation in RNA expression reflects the functionality of the bound site (e.g. activated promoter leads to upregulation in RNA expression). The above analyses were done considering each patient of the cohort as a biological replica.

### Integration between ChIP Seq and RNA-seq data

The integration analysis for ChIP Seq and RNA-seq was performed using the R package Rcade. The differentially expressed (DE) RNA were obtained using R package limma, with an absolute fold change greater than 2.0 and a *p*-value lower than 0.01. In-house Python code (Python 3.7) was used for visualisation [92]. We selected regions that were annotated as enhancers and were intronic and distal intergenic regions, which represent majority of enhancer regions [57]. Enhancers within 50 kb of a TSS were shown to act directly on the nearest gene [57] and, thus, we linked these proximal enhancers with the nearest gene that was within 50 kb. These enhancer-associated genes were integrated with DE genes. The integrated gene sets enriched biological pathways were assessed using the clusterProfiler package in R. A *p*-value < 0.05 was set to select enriched pathways in the Gene Ontology (GO) and Kyoto Encyclopeida of Genes and Genomes (KEGG) databases.

### ChromHMM fragmentation

ChromHMM (v1.18) was used to train and annotate chromatin states [93]. Binarised input files were generated, using the BinarizeBed command, from.bed files of each of the 4 histone marks all GICs and separately all iNSCs. Binarised files were then used as input for the LearnModel command. In brief, the LearnModel command was run separately on iNSC and GIC ChIP Seq data and for a different number of states—the number of states tested for was 4, 6, 8, 10, 12, 14 and 16. Emission parameters of the trained models were compared in order to select a number of states which produced known chromatin states, for instance: quiescent, active transcription, enhancer, repressed polycomb. The number of states selected was 8. Once an appropriate number of states were selected, models trained on iNSC and GIC were compared to evaluate which model best generalised to both cell types, it was found that both models generalised similarly well; however, the iNSC trained model was used in segmentation of samples. Using the selected model, all GIC data merged together, all NSC data merged together and each GIC and NSC line individually, were segmented and the enrichment of each chromatin state determined using the MakeSegmentation and OverlapEnrichment commands. Finally, the enrichment of each state relative to the TSS and TES were determined using the NeighborhoodEnrichment command. Segmented peaks were annotated in R (v4.2) using the ChIPSeeker package (v1.34.1) [94].

### ChIP coverage heatmaps

ChIP coverage heatmaps were generated for GIC and NSC ChIP Seq data using the deeptools (v3.5.2) package [87]. For each ChIP marker, bigwig files for each GIC sample were averaged using the bigwigAverage function. Next, signal distribution was computed using the computeMatrix and the scale-regions option. Other options specified were to set the before region start length and after region start length to 3000 bp, bin size was set to 100 bp, score type was set to “mean” and region body length was set to 1000.

### Gene set analysis

G profiler tool was [95] used to assess biological pathways terms that showed significant enrichment in the various gene sets. The enrichment for each term was deemed statistically significant if the adjusted *p*-value (FDR) was lower than 0.05. Cytoscape v.3.7.215 was used to visualise relevant biological networks of enriched pathways, together with EnrichmentMap application. Several layout parameters were tuned to achieve the current Cytoscape visualisation.

## Supplementary Information

Additional file 1: Figures S1-S12. Figure S1- Single track analysis of the ChIP Seq dataset to identify outliers. a) Schematic of the experimental workflow. EPSC: expanded potential stem cells, iNSC: induced neural stem cells, GIC: glioblastoma initiating cells. b) Number of ChIP Seq peaks found in the 10 GIC (top panel) and in the 10 iNSC (bottom panel) for the four HM. Arrows and italic bold font show a lower number of peaks for H3K36me3 GIC54 and H3K27me3 GIC61. c) Person correlation heatmap represents the 40 ChIP Seq tracks for the four HM of the ten patients. Red font highlights GIC19 H3K27ac clustering apart from other H3K27ac tracks. The two samples identified in Fig. S1b with a low number of peaks are shown in italic. Figure S2- Complete dataset used in the study. a) Person correlation heatmap represents the complete ChIP Seq tracks used in this study including the four HM of the patients’ GIC. b) Person correlation heatmap represents the complete ChIP Seq tracks used in this study including the four HM of the patients’ iNSC. Figure S3- Comparative analysis of iNSC and GIC for each HM at peak level. a) Principal component analysis (PCA) representing affinity sites in GIC and iNSC for the four histone modifications (H3K4me3, H3K36me3, H3K27ac and H3K27me3). b) Correlation heatmaps representing significantly differentially bound sites (FDR<0.05) between the GIC (purple) and iNSC (pink) for the four histone modifications across genome. c) Venn diagram showing the overlapping peaks for each HM. Figure S4- Comparative analysis of iNSC and GIC for each HM at gene level. a-b) Visualization of pathways enriched from genes uniquely found linked to activating (a) and repressing (b) HM in GIC as compared to iNSC. Pathways are annotated based on pathway enrichment analysis performed with Reactome and represented as circle, colours represent each histone (see legend), size of the circle is proportional to the number of genes involved in the pathway, all pathways with FDR<0.05 are shown. Figure S5- ChIP and RNA Seq datasets integration and pathway analysis. a) Pearson correlation heatmap of ChIP Seq dataset for each histone modifications and RNAseq dataset [23] integrative analysis for all genes in GIC (purple square) and iNSC (pink square). RNAseq data are represented as log fold change of Differentially Expressed (DE) genes between GIC and iNSC. LogFC DE>1 and <-1 for genes up and downregulated in GIC as compared to iNSC respectively. LogFC DE>1 and <-1 when genes are down and upregulated in iNSC as compared to GIC respectively. b-c) Volcano plot representing genes bound by each histone modification based on the fold change on the input. Among them, the upregulated genes (LogFC(DE)>1) are shown in red and the downregulated (LogFC (DE)<-1) are shown in blue in GIC (b) and iNSC (c). d) Venn diagram showing percentages of specific and common genes in GIC (purple) and iNSC (pink) identified by peak calling for each histone modification. e) Visualisation of the enriched pathways identified in GIC from genes upregulated in GIC as compared to iNSC and associated with a gain of activating HM (H3K4me3, H3K36me3 and H3K27ac) and a loss of repressing HM (H3K27me3). Pathways are annotated based on pathways enrichment analysis performed with Reactome and represented as circle, colours represent each HM (see legend), size of the circle is proportional to the number of genes involved in the pathway (FDR<0.05). Figure S6- Pathways analysis and identification of *GSC* as differentially regulated and DE gene. a) Visualisation of the enriched pathways identified in GIC from genes downregulated in GIC as compared to iNSC and associated with the loss of activating HM and gain of repressing HM. Pathways are annotated based on pathways enrichment analysis performed with Reactome and represented as circle, colours represent each HM (see legend), size of the circle is proportional to the number of genes involved in the pathway (FDR<0.05). b) Comparison of Chip Seq tracks of each modification in iNSC (pink) and GIC (purple) in the GSC locus. Tracks shown are an average of the 10 patients and the black square highlights the region with differential peaks. c-d) Representative images of GSC protein expression (green) in iNSC and GIC of patients 19 and 61 (c). Nuclei are countered staining with DAPI. Negative control shows the immunofluorescence without primary GSC antibody (d). Scale bars: 50µm (c)and 100µm (d). e) GBMap [97] analysis showing the expression of *GSC* in the different cell types of the brain. Figure S7- ChromHMM analysis. a) Percentages of overlap of the two models (trained on iNSC or on GIC) in GIC and iNSC. b) Heatmap of the emission parameters displaying the overlap fold enrichment for various external genomic annotations. c) Distribution of ChIP Seq peaks’ genomic annotations for each chromatin state in GIC and iNSC. Heatmap represent Fisher’s exact test statistical analysis and fold change of ChIP Seq peaks between GIC and iNSC. d) Venn diagram showing number of peaks specific to GIC (purple), overlapping with iNSC and specific to iNSC (pink) for each chromatin state defined by ChromHMM analysis. Figure S8- ChromHMM analysis without and with RNA Seq integration. a) Percentages of upregulated (red) and downregulated (blue) genes in GIC as compared to iNSC (left) and in iNSC as compared to GIC (right) in each chromatin state based on transcriptomic dataset from the SYNGN Cohort. Number of genes is also specified for each condition. b) Visualisation of the enriched pathways identified in GIC from genes upregulated in GIC as compared to iNSC and associated with a gain of activating chromatin state. Pathways are annotated based on pathways enrichment analysis performed with Reactome and represented as circle, colours represent each histone (see legend), size of the circle is proportional to the number of genes involved in the pathway (FDR<0.05). c) mRNA expression of *OAS1* and *OAS3* in iNSC and GIC from the RNAseq dataset of the SYNGN cohort [23]. Results are expressed in log 2 (tpm) transcript per million (tpm). One-way ANOVA test.*p value<0.05, **p value<0.01 and ***p value<0.001. d) Survival curve of primary glioblastoma patients with high and low expression of *OAS1* (left panel) and *OAS3* gene (right panel). Source: TCGA. Stat test: log-rank,*p value<0.05, **p value<0.01 and ***p value<0.001. e) Spatial expression of *OAS1* (middle) and *OAS3* (right) in glioblastoma bulk samples, analysed on IvyGap. The left panel shows an example of histological anatomic structure identified in a sub-block and the right panel represents the expression of *OAS1* and *OAS3* in RNAseq data from anatomic structures shown as log2 normalised gene expression. Leading Edge is defined as the border of the tumour, where ratio of tumour to normal cells is 1-3 / 100. Infiltrating tumour is defined as the intermediate zone between leading edge and cellular tumour, where ratio of tumour to normal cells is 10-20 /100. Cellular tumour is defined as tumour core, where tumour to normal cells is 100-500 / 1. One-way ANOVA test.*p value<0.05, **p value<0.01 and ***p value<0.001. f) Single cell RNAseq data from [31] showing *OAS1 *(top) and *OAS3* (bottom) expression (left panels) in scRNAseq-defined clusters in glioblastoma samples (right panels). Data are plotted as tSNE, with logTPM expression ranging from light orange to dark. Figure S9- ChIP and RNA Seq integration and identification of differentially regulated and DE genes. a) Visualisation of the enriched pathways identified in GIC from genes downregulated in GIC as compared to iNSC and associated with the loss of activating HM and gain of repressing HM. Pathways are annotated based on pathways enrichment analysis performed with Reactome and represented as circle, colours represent each HM (see legend), size of the circle is proportional to the number of genes involved in the pathway (FDR<0.05). b) Survival curve of primary glioblastoma patients with high and low expression of *UBE2D*. Source: TCGA. Stat test: log-rank, *p value<0.05, **p value<0.01 and ***p value<0.001. c) Single cell RNAseq data from [31] showing *UBE2D* expression in scRNAseq-defined clusters in glioblastoma samples (right panels). Data are plotted as tSNE, with logTPM expression ranging from light orange to dark. d) Bar plot showing the down and upregulated genes associated with proximal enhancers only found in GIC and in iNSC. Stat test: Fisher’s exact test,***p value<0.001. e) Gene ontology analysis showing the biological processes regulated by the upregulated genes associated with proximal enhancers found only in GIC (left) and downregulated genes associated with proximal enhancers found only in iNSC (right). Enrichment analysis was performed with GO Biological Processes, with an adjusted *p *value < 0.05. Figure S10- Switching states as assessed by ChromHMM. a) Heatmap showing the number of peaks changing from each NSC state to each GIC state in at least 2 patients. b) Pie charts show percentages of peaks in each transitioning states between in GIC and iNSC. c) Distribution of genomic annotations of peaks switching from repressing in iNSC to activating state in GIC (top plots) and from activating in iNSC to repressing switches in GIC (bottom plots). Figure S11- SMOX is a novel target gene identified in transitioning states. a) Comparison of ChIP Seq tracks of each modification from iNSC (pink) and GIC (purple) in the SMOX locus. Tracks shown are an average of the 10 patients and the black squares highlight the regions with differential peaks. b) Survival curve of primary glioblastoma patients with high and low expression of *SMOX*. Source: TCGA. Stat test: log-rank, *p value<0.05, **p value<0.01 and ***p value<0.001. c) Spatial expression of *SMOX* in glioblastoma bulk samples, analysed on Ivy -GAP. The left panel shows an example of histological anatomic structure identified in a sub-block and the right panel represents the expression of *SMOX *in RNAseq data from anatomic structures shown as log2 normalised gene expression. Leading Edge is defined as the border of the tumour, where ratio of tumour to normal cells is 1-3 / 100. Infiltrating tumour is defined as the intermediate zone between leading edge and cellular tumour, where ratio of tumour to normal cells is 10-20 /100. Cellular tumour is defined as tumour core, where tumour to normal cells is 100-500 / 1. One-way ANOVA test. *p value<0.05, **p value<0.01 and ***p value<0.001. d) Single cell RNAseq data from [31] showing *SMOX* expression (top panel) in scRNAseq-defined clusters in [31] (bottom panel). Data are plotted as tSNE, with logTPM expression ranging from light orange to dark. e) GBMap [97] analysis showing the expression of *SMOX* in the different cell types present in the brain. f) mRNA expression of the *SMOX* in iNSC and GIC from three patients of the SYNGN cohort (19, 52 and 61) measured by RTqPCR. Results are expressed in two delta Ct (detailed in Material and Methods section). g) Representative images of SMOX staining with immunofluorescence (green) in iNSC and GIC of patients 19 and 61. Nuclei are counterstained with DAPI. Scale bar:100µm. h) Cell viability measured with CellTiter Glo Promega Kit in response to increasing doses of SMOX inhibitor MDL72527 (1, 10, 100µM). Results are expressed in fold change of the average from three patients (19, 52 and 61) standardised on the vehicle (0) for iNSC (pink border) and GIC (purple border). i) Representative images of GIC nuclei, counterstained with DAPI, on the bottom side of the transwell membrane after 24 hours of treatment with SMOX inhibitor MDL72527 (10µM). Scale bar:100µm. Figure S12- GABBR2 is a novel target gene identified in transitioning states. a) Comparison of ChIP Seq tracks of each modification from iNSC (pink) and GIC (purple) in the GABBR2 locus. Tracks shown are an average of the 10 patients and the black square highlights the region with differential peaks. b) mRNA expression of the *GABBR2* in iNSC and GIC from three patients of the SYNGN cohort (19, 52 and 61) measured by RTqPCR. Results are expressed in two delta Ct (detailed in Material and Methods section). c) Survival curve of primary glioblastoma patients with high and low expression of *GABBR2*. Source: TCGA. Stat test: log-rank,*p value<0.05, **p value<0.01 and ***p value<0.001. d) Spatial expression of *GABBR2* in glioblastoma bulk samples, analysed on Ivy -GAP. The left panel shows an example of histological anatomic structure identified in a sub-block and the right panel represents the expression of *GABBR2 *in RNAseq data from anatomic structures shown as log2 normalized gene expression. Leading Edge is defined as the border of the tumour, where ratio of tumour to normal cells is 1-3 / 100. Infiltrating tumour defined as the intermediate zone between leading edge and cellular tumour, where ratio of tumour to normal cells is 10-20 /100. Cellular tumour defined as tumour core, where tumour to normal cells is 100-500/ 1. One-way ANOVA test. *p value<0.05, **p value<0.01 and***p value<0.001. e) Single cell RNAseq data showing *GABBR2* expression (left panel) in scRNAseq of glioblastoma samples in clusters defined in [31] (right panel). Data are plotted as tSNE, with logTPM expression ranging from light orange to dark. f) GBMap analysis showing the expression of *GABBR2 *in the different cell types present in the brain. g) Cell viability measured with CellTiter Glo Promega Kit in response to increasing doses of GABBR2 agonist Baclofen (red bars) and the antagonist CGP36742 (blue bars) (0, 10, 100, 200µM). Results are expressed in fold change of the average from three patients (19, 52 and 61) standardised on the vehicle (0) for iNSC (pink border) and GIC (purple border). h) Representative images of GIC nuclei, counterstained with DAPI, on the bottom side of the transwell membrane after 24 hours of treatment with GABBR2 agonist Baclofen (100µM) and the antagonist CGP36742 (200µM). Scale bar:100µm.

Additional file 2: Table S1: Detailed information of data used for the generation of the cytoscapes Additional file 1: Fig. S4.

Additional file 3: Table S2 Detailed information of data used for the generation of the cytoscapes Additional file 1: Fig. S6.

Additional file 4: Table S3: Detailed information of data used for the generation of the cytoscapes Additional file 1: Fig. S8.

Additional file 5: Table S4 Detailed information of data used for the generation of the cytoscapes Fig. 3.

Additional file 6: Table S5 Detailed information of data used for the generation of the pie chart Fig. 4a.

## Data Availability

All data generated or analysed during this study are included in this published article and its supplementary information files. The ChIP-seq data are available in the NCBI Gene Expression Omnibus with accession number GSE259262 [24] Publicly available datasets used in the study include GSE155994, TCGA datasets (dbGaP Study Accession: phs000178 [96]) analysed with GlioVis [29] GSE107560 (analysed with IvyGap [30]). Datasets used by GBmap [97] can be accessed under GEO/EGA/SRA accession numbers: GSE103224, GSE131928, GSE138794 (EGAS00001002185, EGAS00001001900, and EGAS00001003845), GSE148842, GSE139448, EGAS00001004422, PRJNA579593, GSE117891, GSE157424, EGAS00001004656, EGAS00001005300, GSE84465, PRJNA588461, GSE135437, GSE163108, and GSE163120.In-house Python codes are available from Zenodo [92] 10.5281/zenodo.14186449.

## Abbreviations

ACTB: Actin Beta
ALK: Anaplastic Lymphoma Kinase
ANXA2R: Annexin A2 Receptor
ATP5B: ATP Synthase, H + Transporting, Mitochondrial F1 Complex, Beta Polypeptide
ChIP Seq: Chromatin Immunoprecipitation Sequencing
CytB: Cytochrome B
DDR: DNA Damage Response
EPSC: Expanded potential stem cells
FDR: False Discovery Rate
FOXM1: Forkhead Box M1
GIC: Glioblastoma initiating cells
GO: Gene Ontology
GPCR: G Protein-Coupled Receptor
GSEA: Gene Set Enrichment Analysis
H3K27ac: Acetylation of Lysine 27 on Histone H3
H3K27me3: Tri-methylation of Lysine 27 on Histone H3
H3K36me3: Tri-methylation of Lysine 36 on Histone H3
H3K4me3: Tri-methylation of Lysine 4 on Histone H3
H3K9ac: Acetylation of Lysine 9 on Histone H3
H3K9me3: Tri-methylation of Lysine 9 on Histone H3
HM: Histone modification(s)
HRR: Homologous Recombination Repair
iNSC: Induced neural stem cells
IvyGAP: Ivy Glioblastoma Atlas Project
KEGG: Kyoto Encyclopedia of Genes and Genomes
MGMT: O6-Methylguanine-DNA Methyltransferase
MYC: Myelocytomatosis oncogene
NSC: Neural stem cells
OAS1/OAS3: 2’-5’-Oligoadenylate Synthetase 1/3
PCA: Principal component analysis
PPARA: Peroxisome Proliferator-Activated Receptor Alpha
qPCR: Quantitative polymerase chain reaction
RFX: Regulatory Factor X
RPL/RPS: Ribosomal Protein Large/Small Subunit
RTK: Receptor tyrosine kinase
RUNX: Runt-Related Transcription Factor
SHH: Sonic Hedgehog
SYNGN: Syngeneic comparison of GIC and iNSC Pipeline
TCGA: The Cancer Genome Atlas
TEM: Transmission electron microscopy
TSS: Transcription start site
UBE2D1: Ubiquitin-conjugating enzyme E2 D1
WNT: Wingless-Related Integration Site
