# Supplementary figures and images for: Mapping chromatin remodelling in glioblastoma identifies epigenetic regulation of key molecular pathways and novel druggable targets
Source: BMC Biol. 2025 Feb 7;23:26. doi: 10.1186/s12915-025-02127-9 (PMC11804007; doi:10.1186/s12915-025-02127-9)

Figure S1

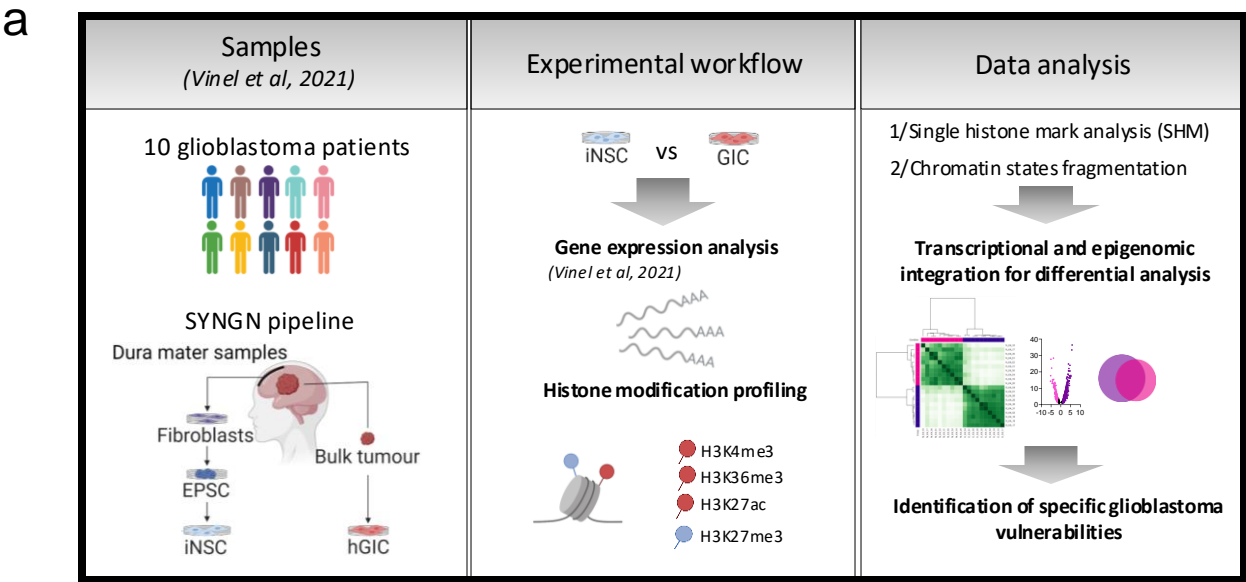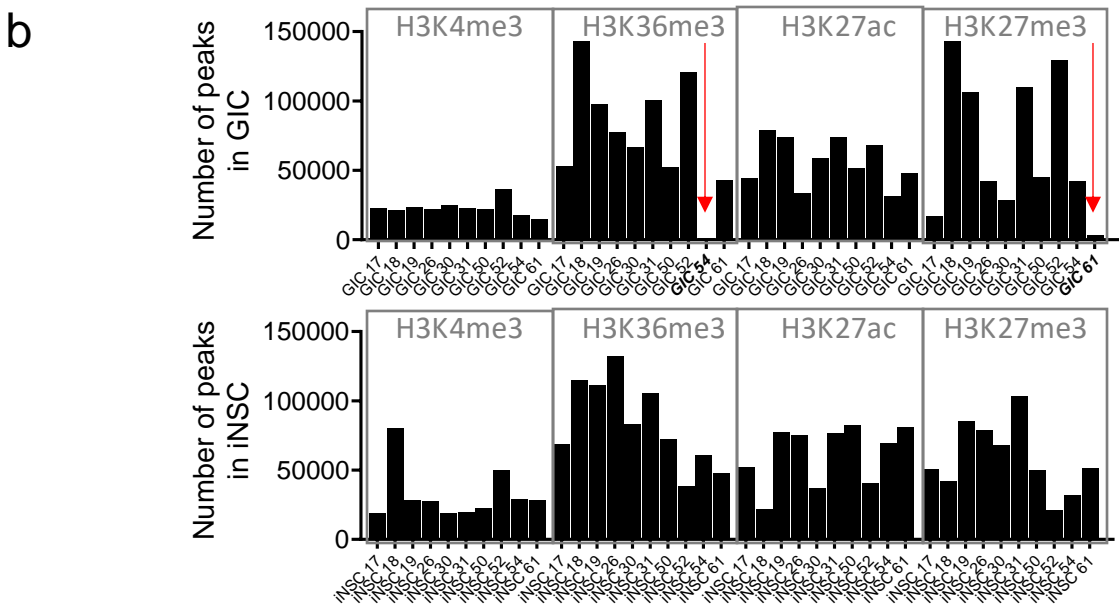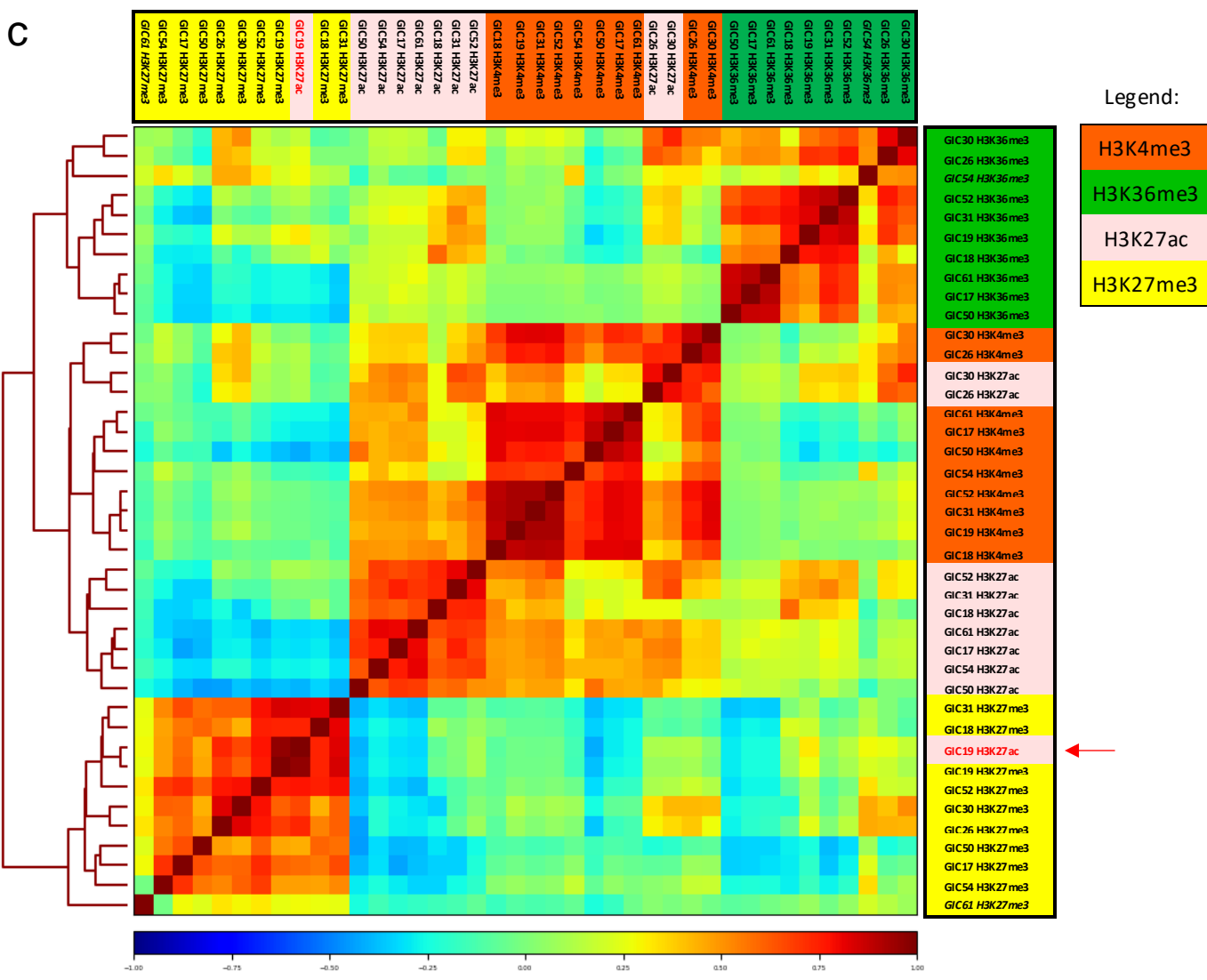

Figure S2

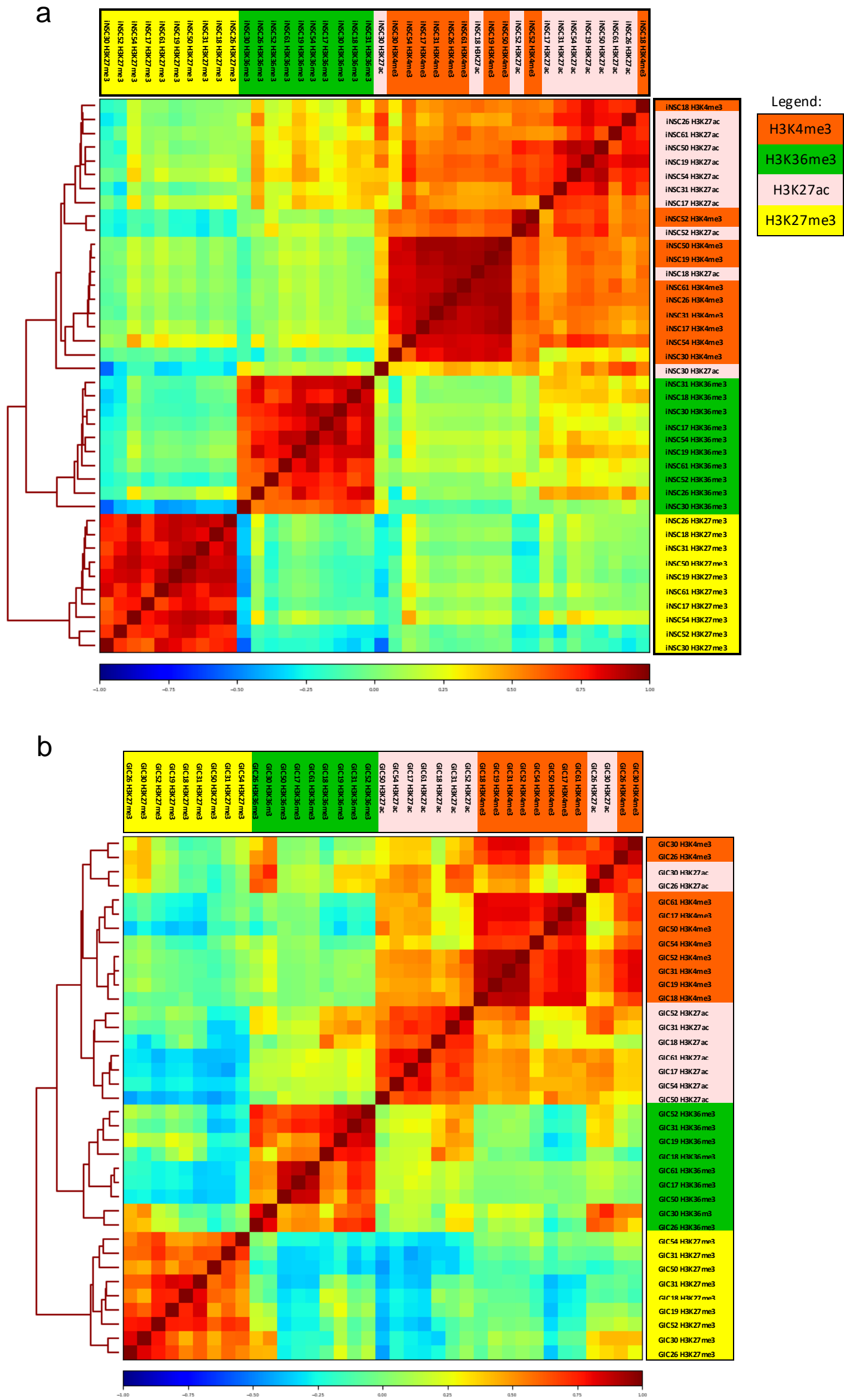

Figure S3

a

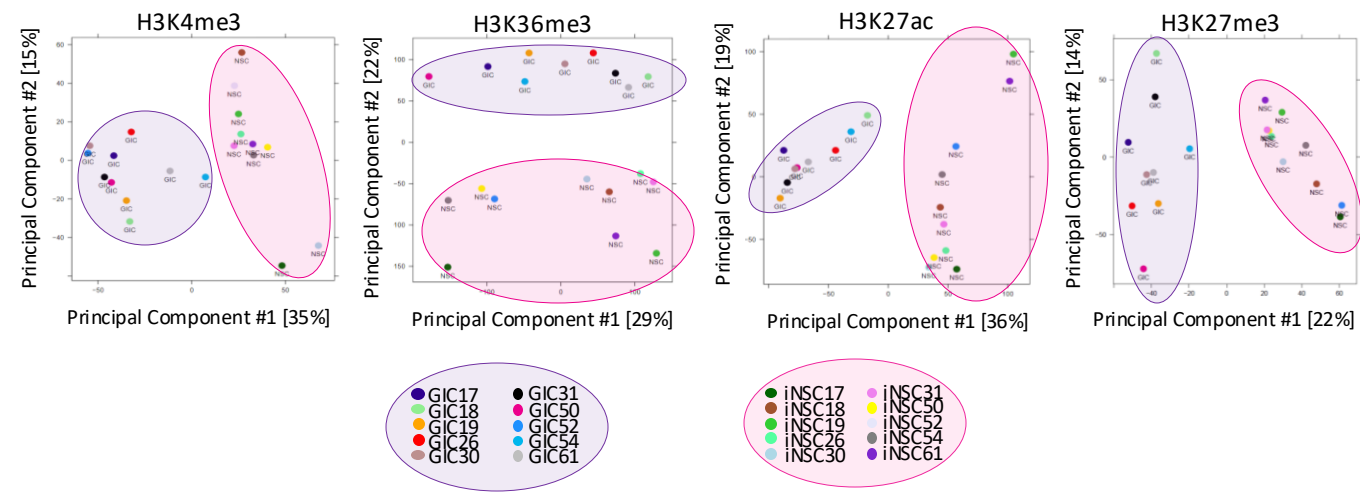

b

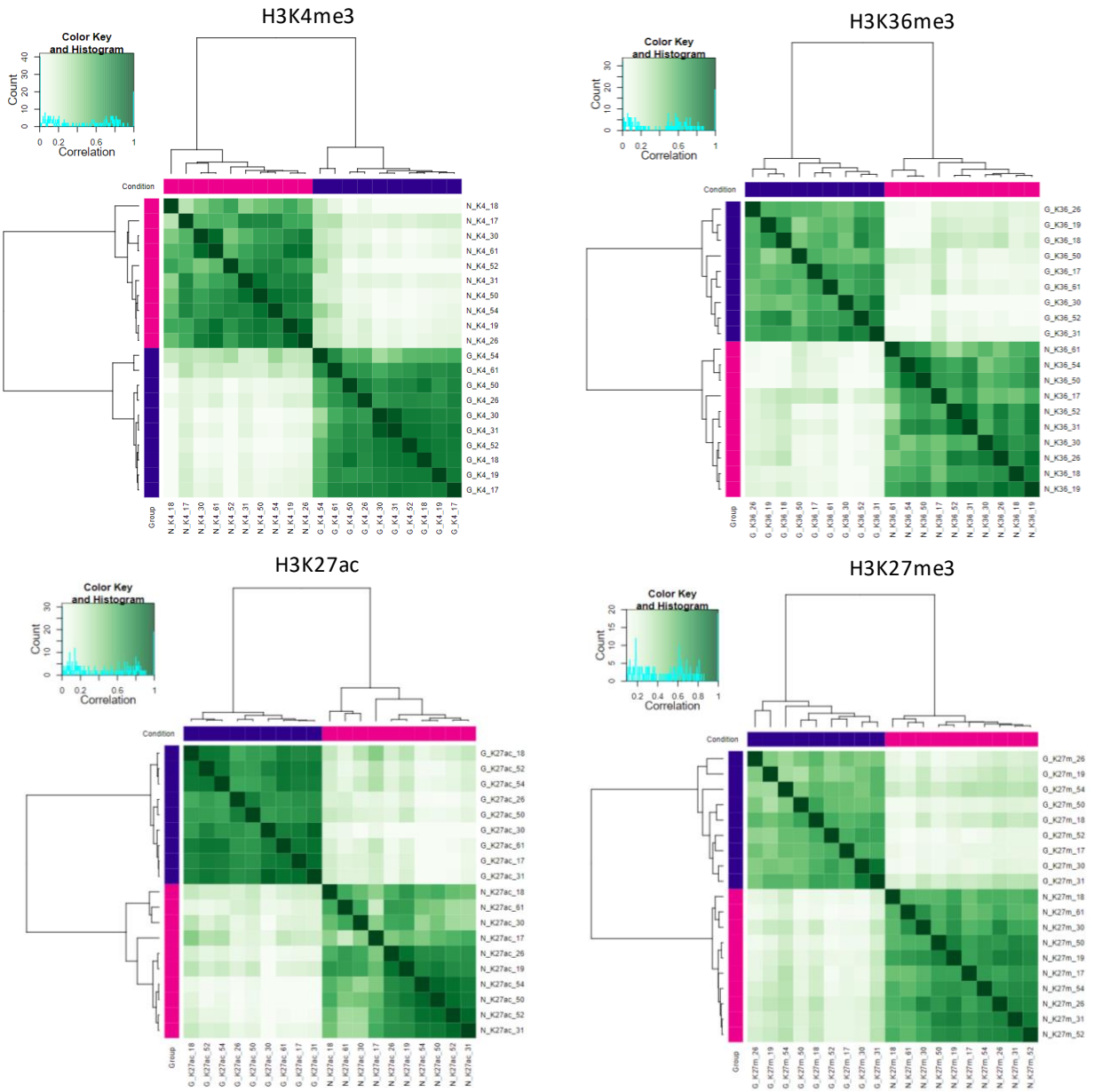

c

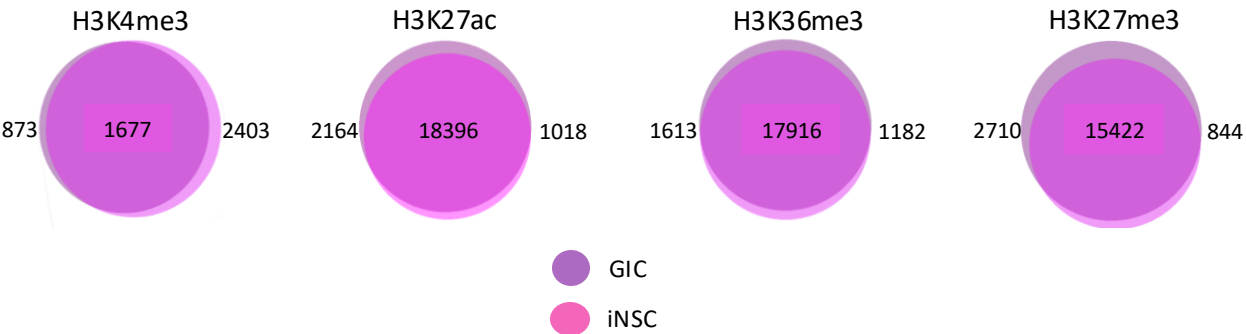

a

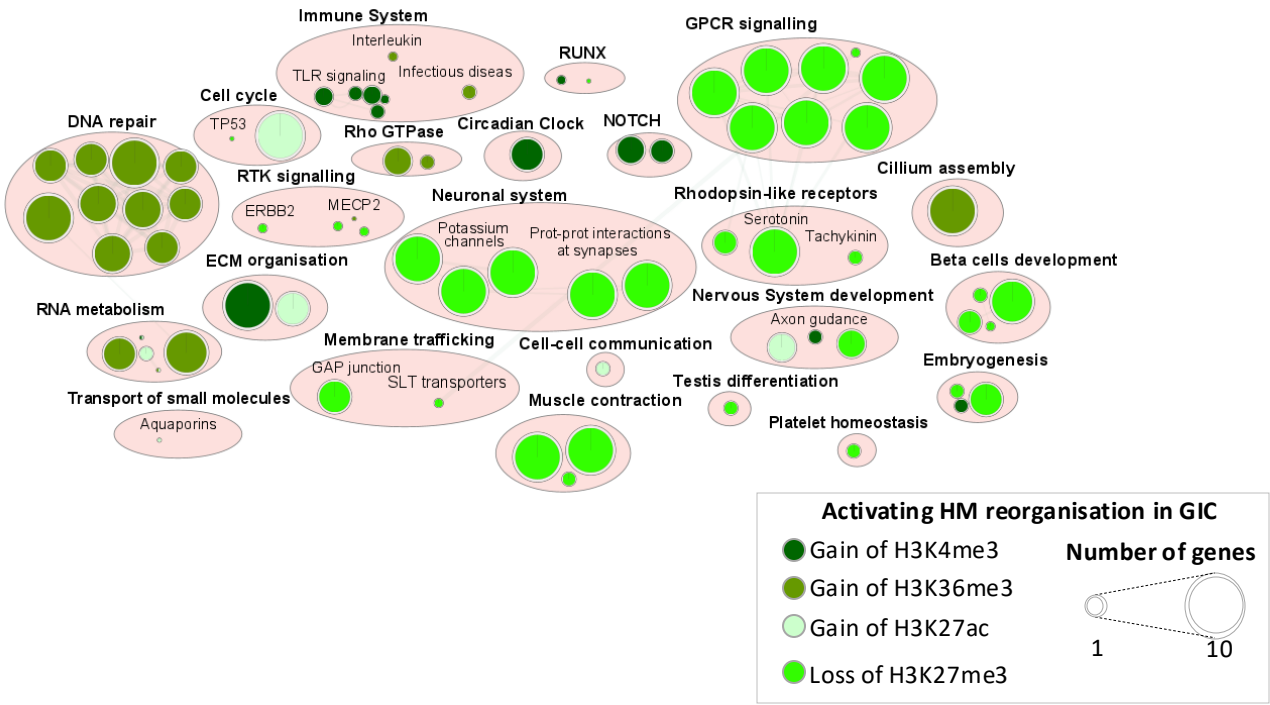

b

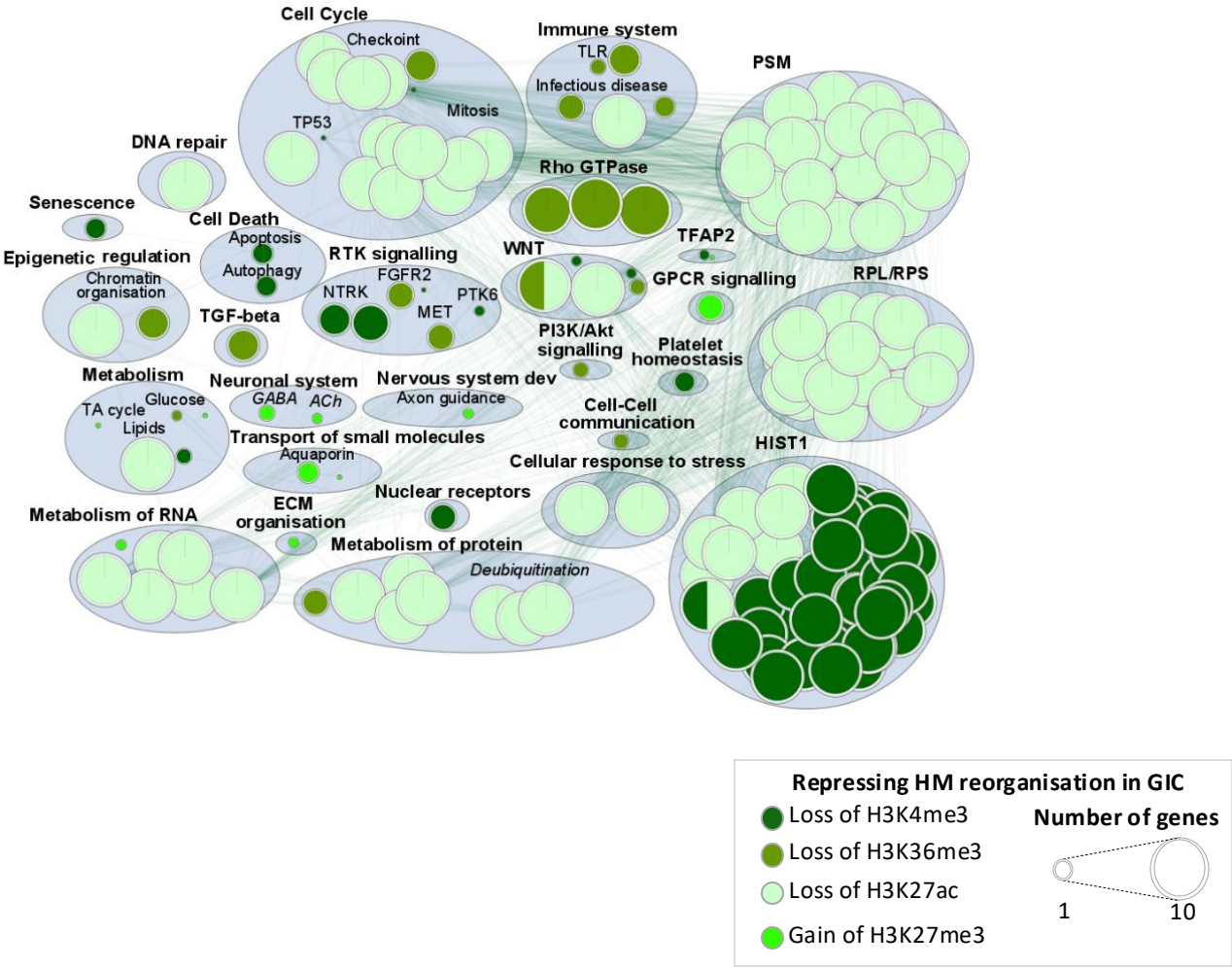

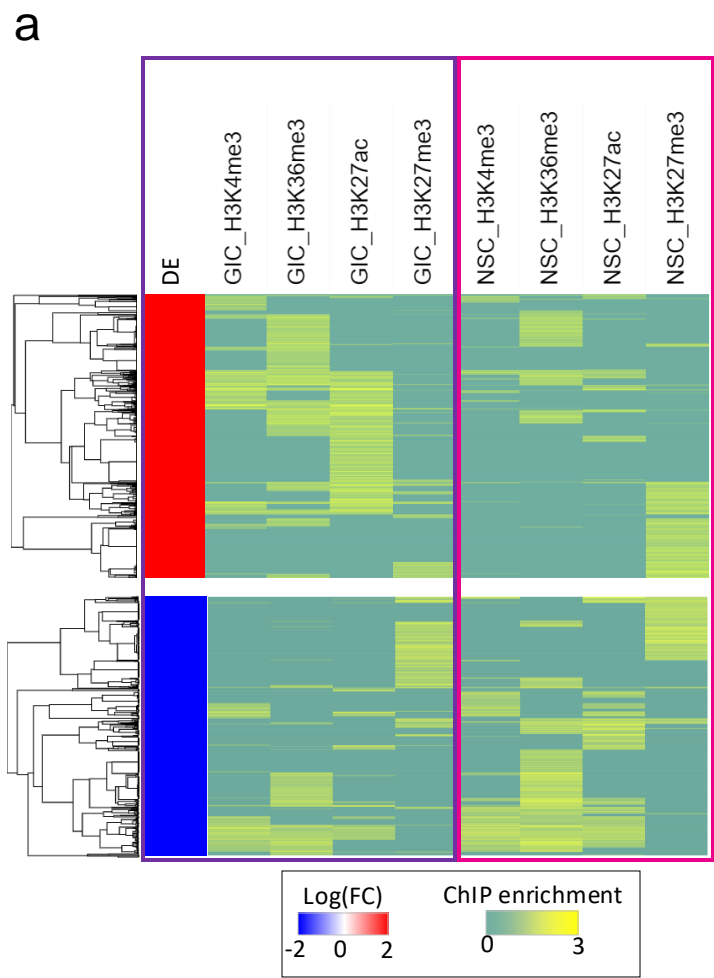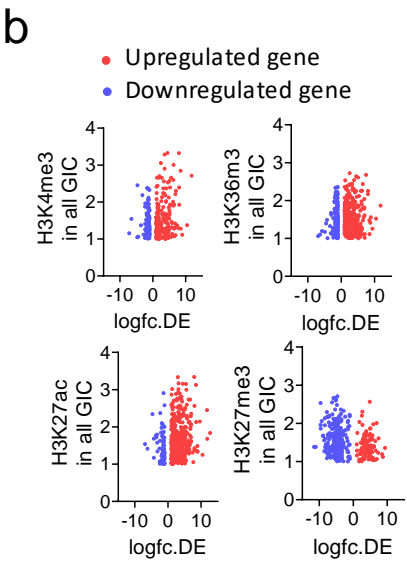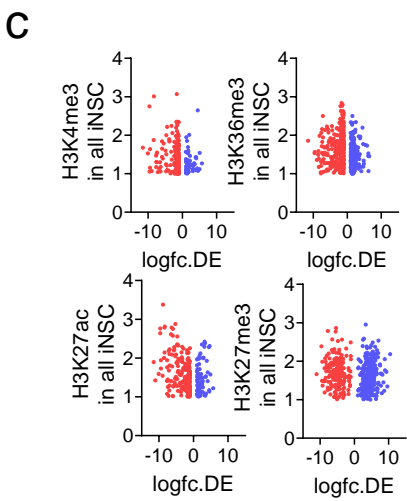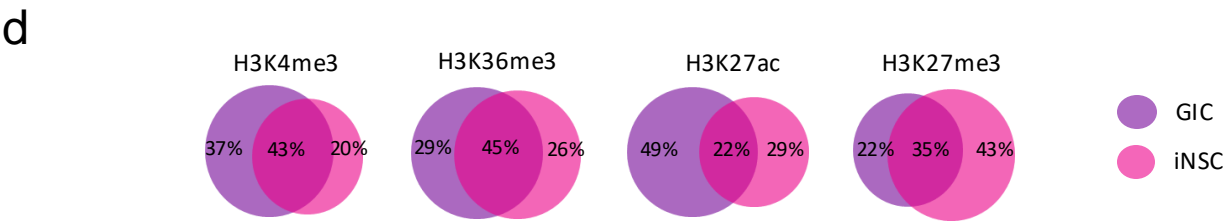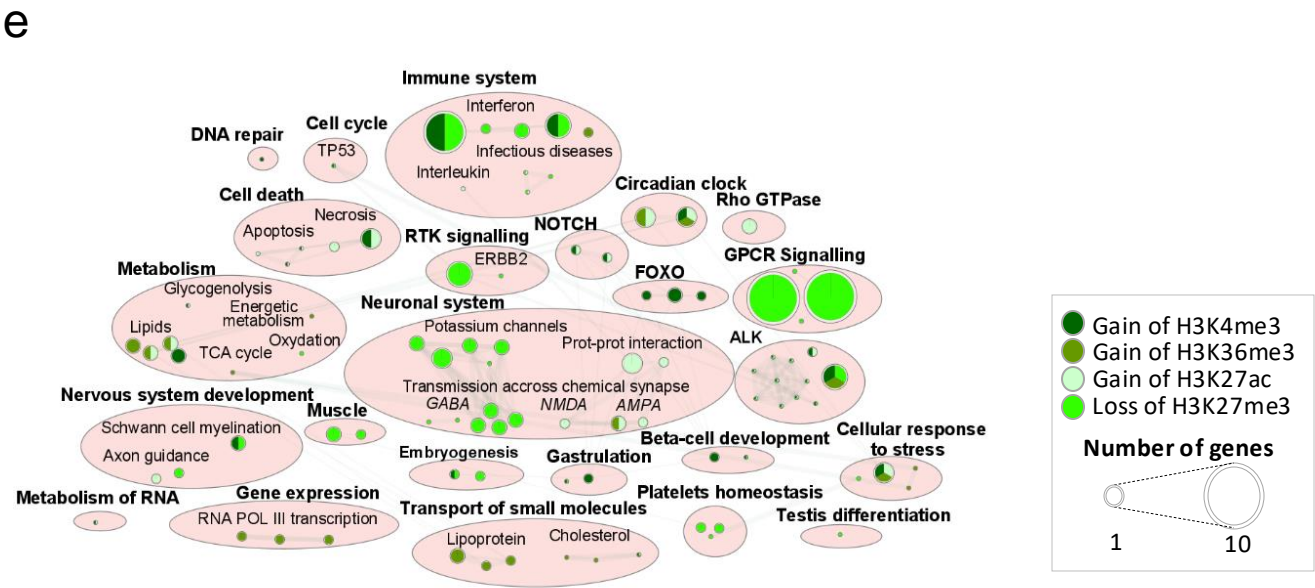

Figure S6

a

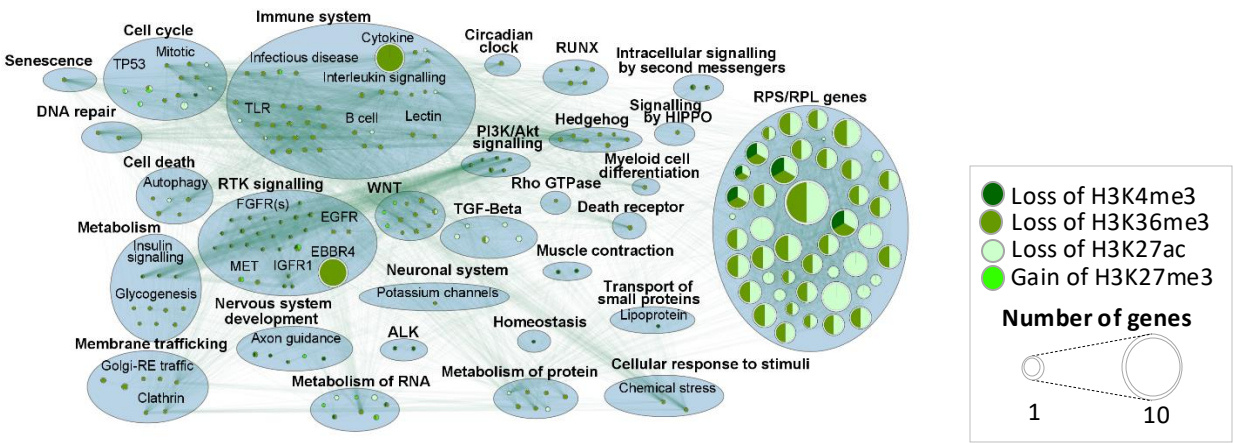

b

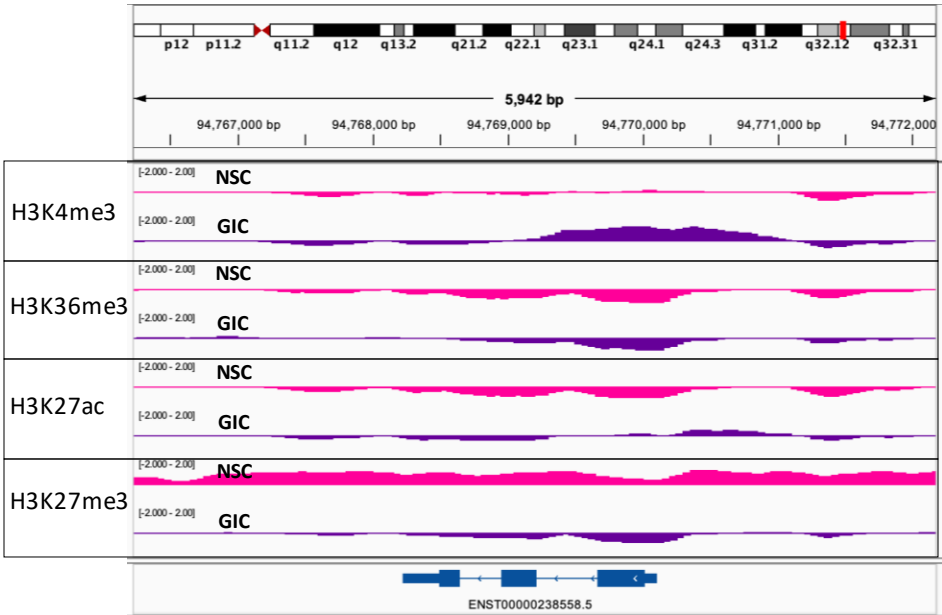

c

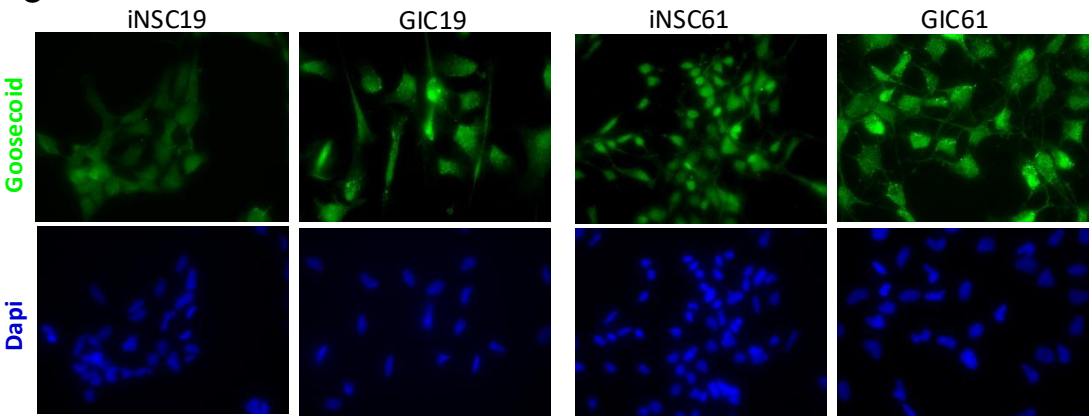

d

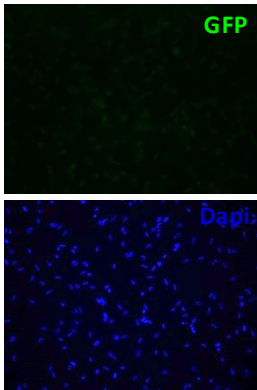

e

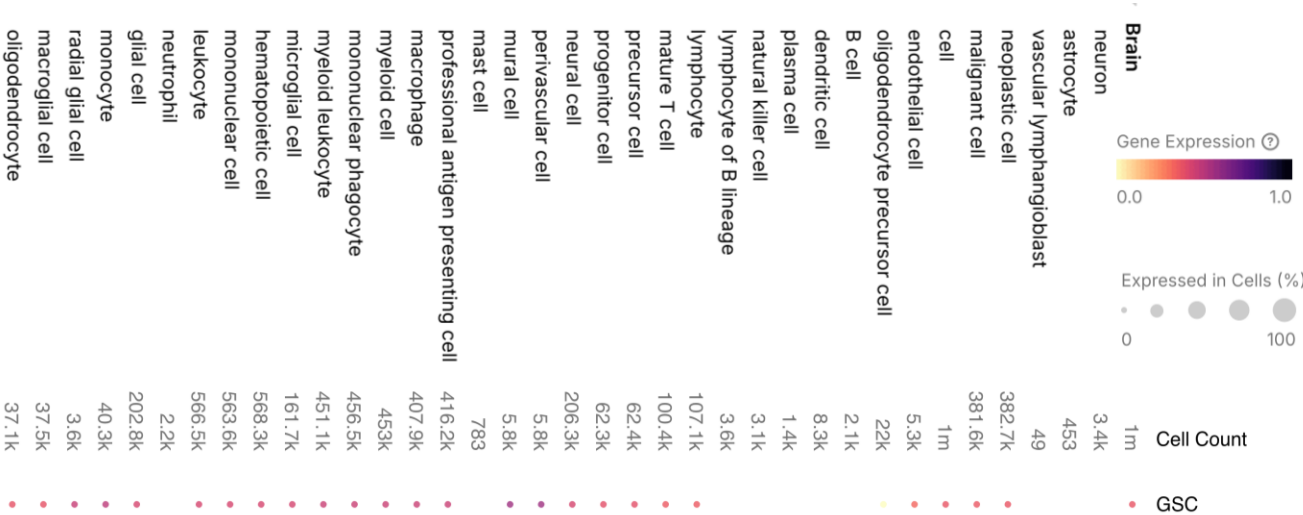

Figure S7

a

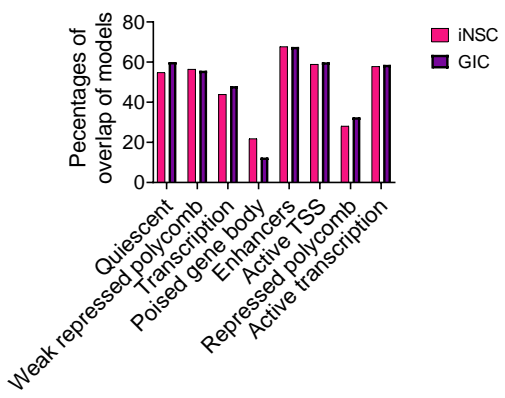

b

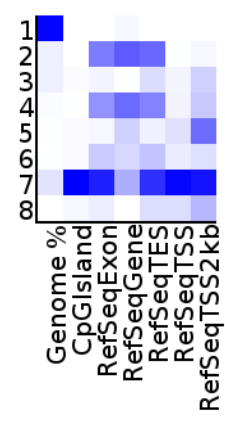

c

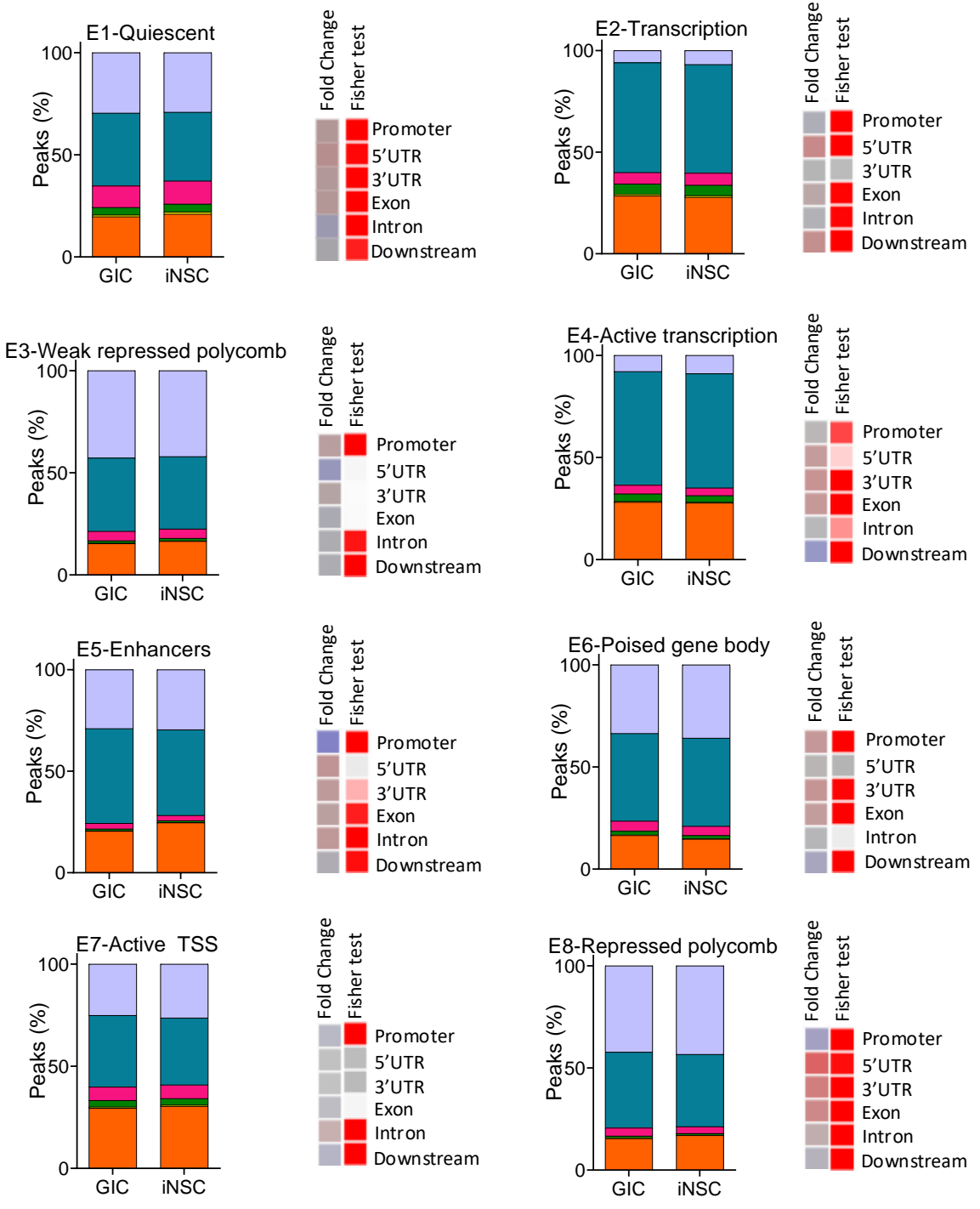

d

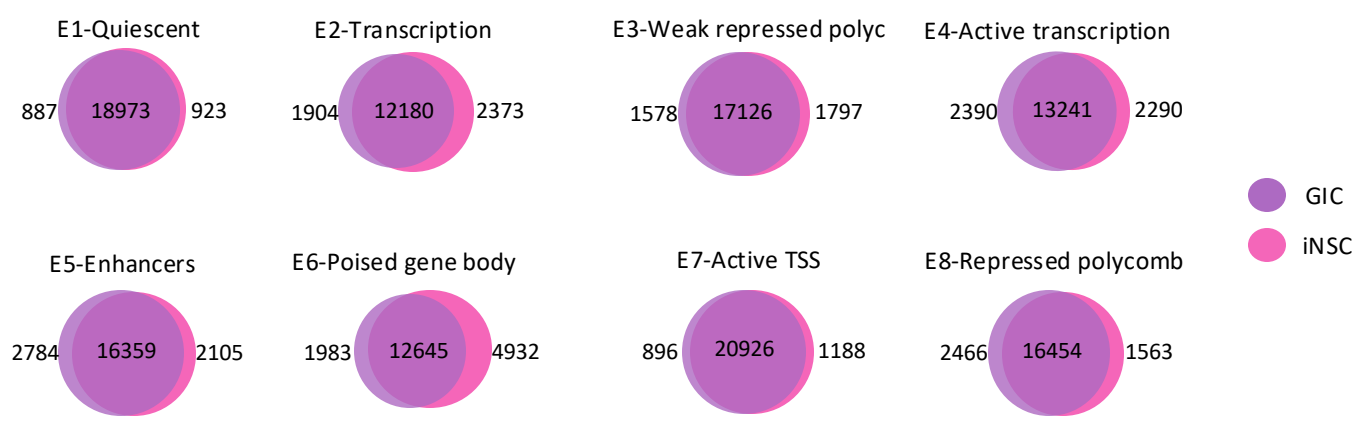

Figure S8

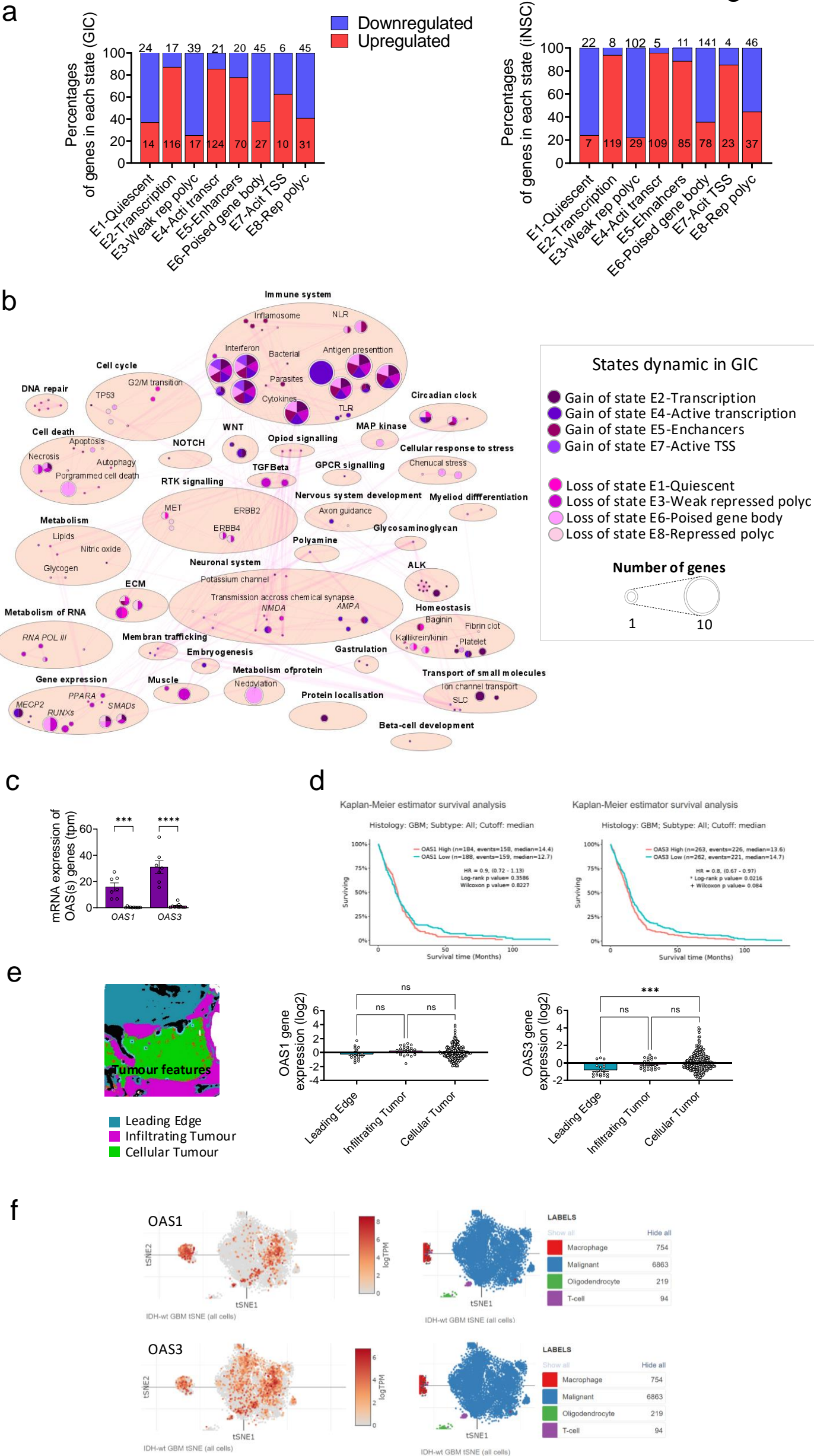

a

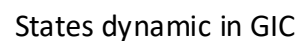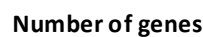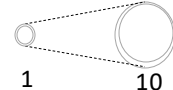

**b**

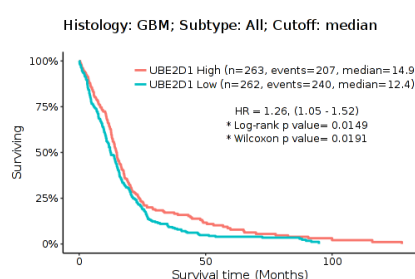

**C**

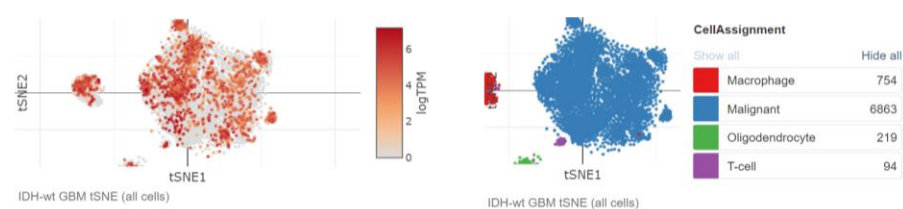

d

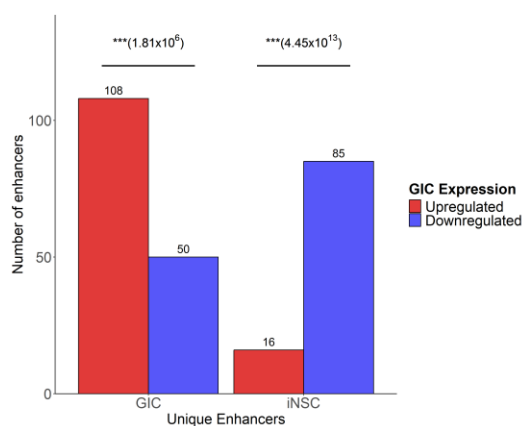

e

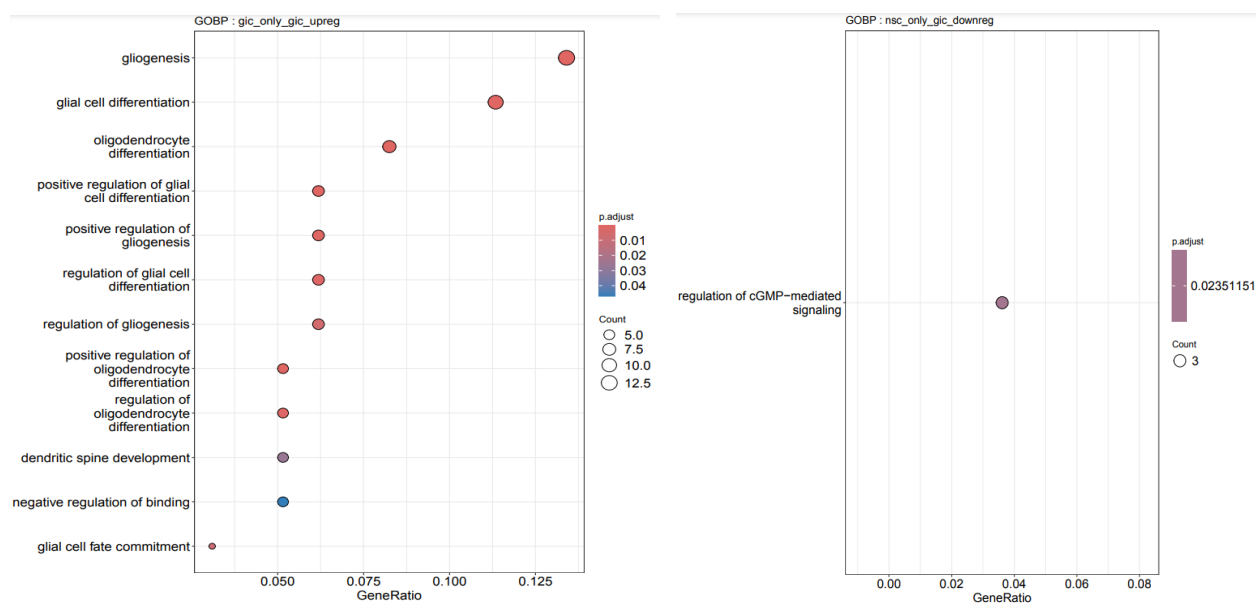

a

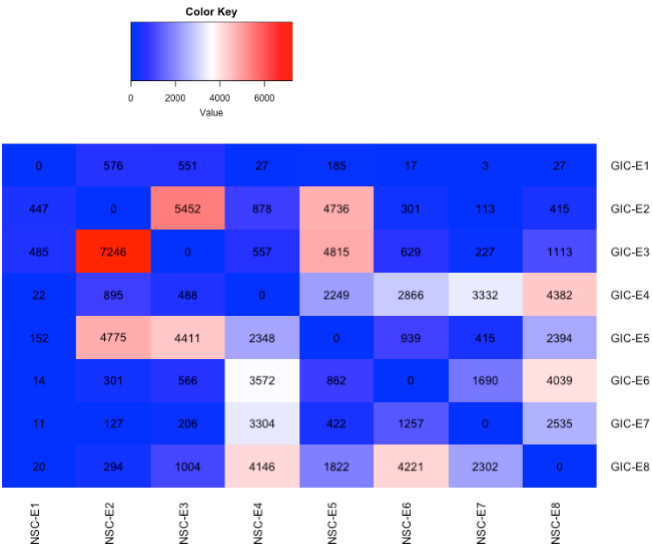

b

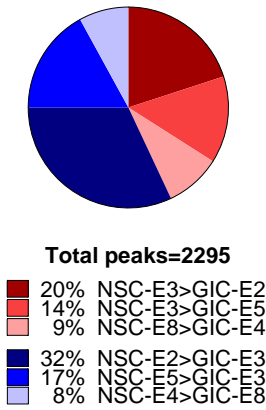

c

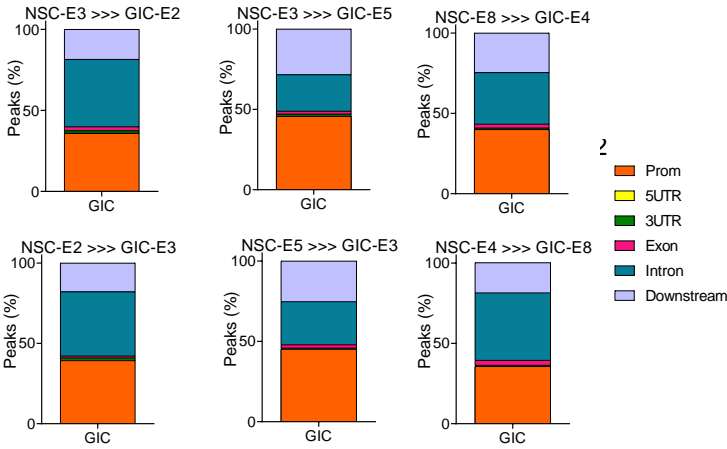

a

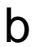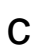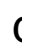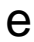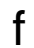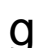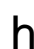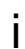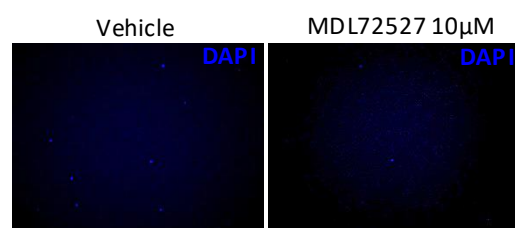

a

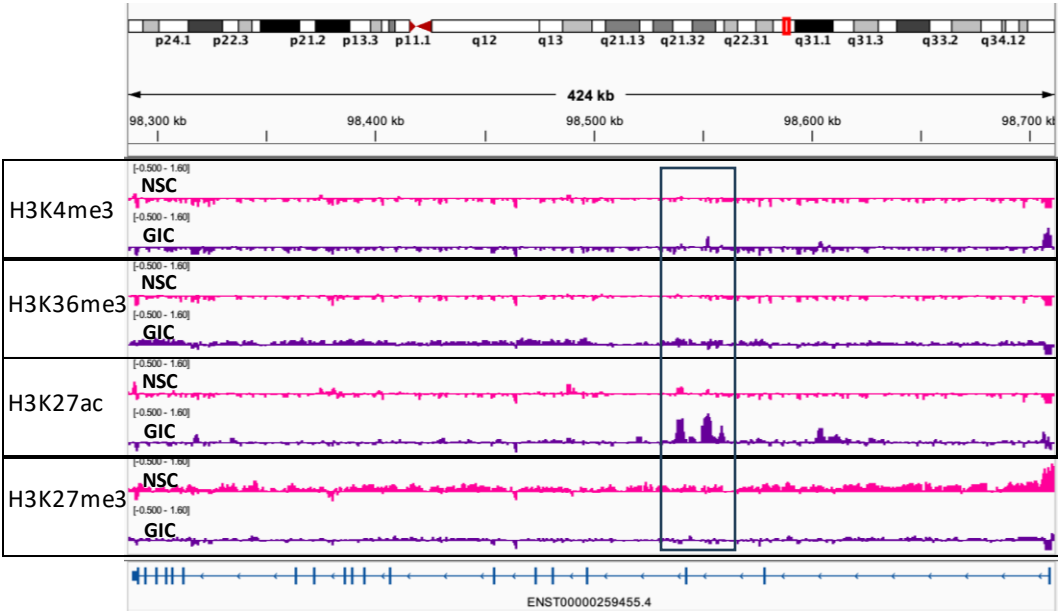

b

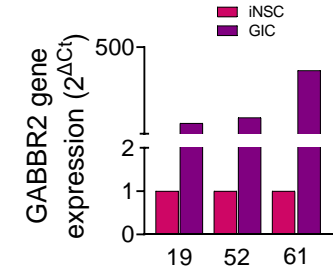

c

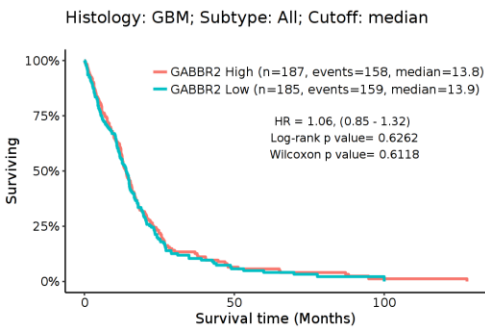

d

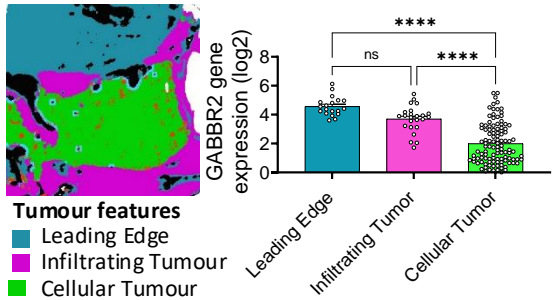

e

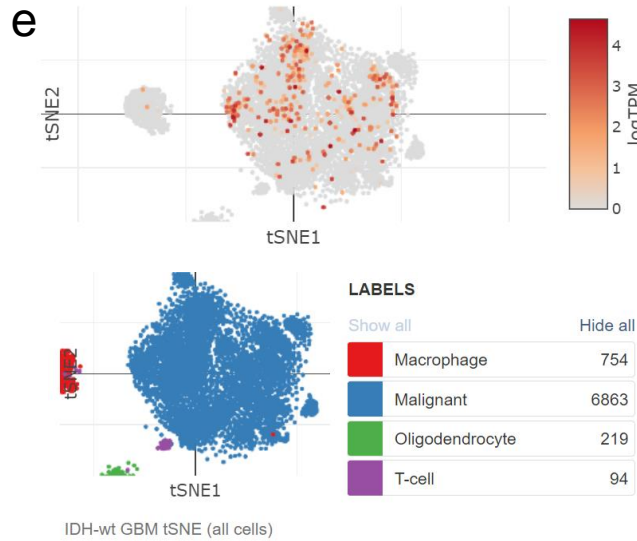

f

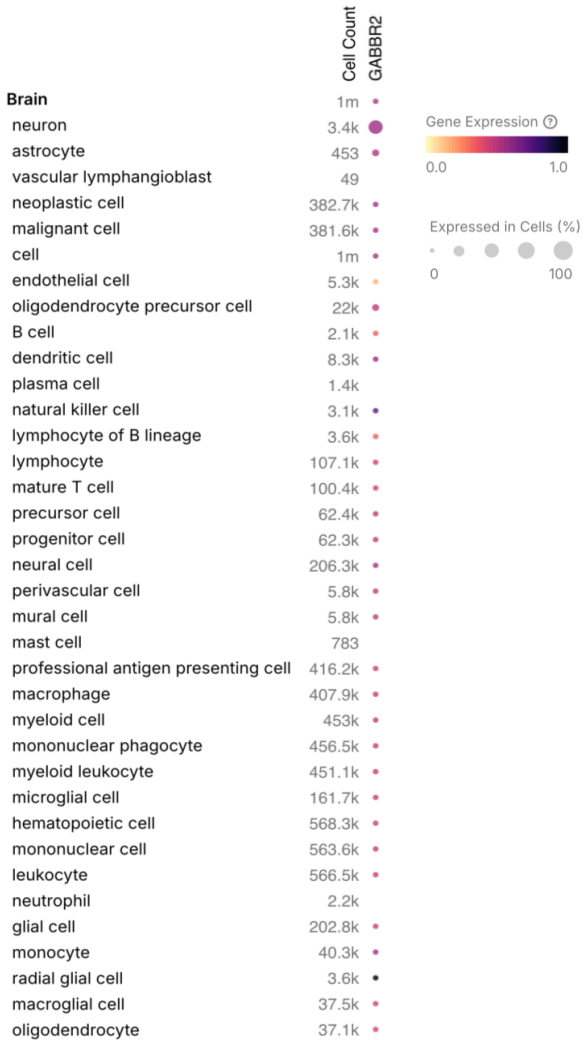

g

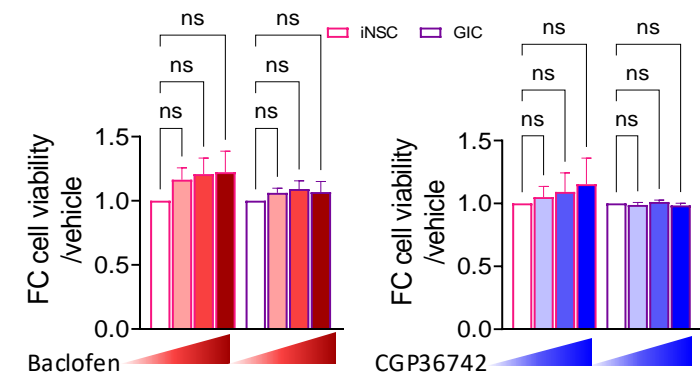

h

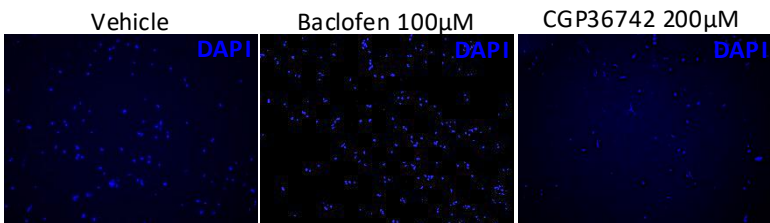

Supplement: Supplementary file 1 — Additional file 1: Figures S1-S12. Figure S1- Single track analysis of the ChIP Seq dataset to identify outliers. a) Schematic of the experimental workflow. EPSC: expanded potential stem cells, iNSC: induced neural stem cells, GIC: glioblastoma initiating cells. b) Number of ChIP Seq peaks found in the 10 GIC (top panel) and in the 10 iNSC (bottom panel) for the four HM. Arrows and italic bold font show a lower number of peaks for H3K36me3 GIC54 and H3K27me3 GIC61. c) Person correlation heatmap represents the 40 ChIP Seq tracks for the four HM of the ten patients. Red font highlights GIC19 H3K27ac clustering apart from other H3K27ac tracks. The two samples identified in Fig. S1b with a low number of peaks are shown in italic. Figure S2- Complete dataset used in the study. a) Person correlation heatmap represents the complete ChIP Seq tracks used in this study including the four HM of the patients’ GIC. b) Person correlation heatmap represents the complete ChIP Seq tracks used in this study including the four HM of the patients’ iNSC. Figure S3- Comparative analysis of iNSC and GIC for each HM at peak level. a) Principal component analysis (PCA) representing affinity sites in GIC and iNSC for the four histone modifications (H3K4me3, H3K36me3, H3K27ac and H3K27me3). b) Correlation heatmaps representing significantly differentially bound sites (FDR<0.05) between the GIC (purple) and iNSC (pink) for the four histone modifications across genome. c) Venn diagram showing the overlapping peaks for each HM. Figure S4- Comparative analysis of iNSC and GIC for each HM at gene level. a-b) Visualization of pathways enriched from genes uniquely found linked to activating (a) and repressing (b) HM in GIC as compared to iNSC. Pathways are annotated based on pathway enrichment analysis performed with Reactome and represented as circle, colours represent each histone (see legend), size of the circle is proportional to the number of genes involved in the pathway, all pathways with FDR [file 12915_2025_2127_MOESM1_ESM.pdf]
